# Structural insights into 2-oxindole-forming monooxygenase MarE: Divergent architecture and substrate positioning *versus* tryptophan dioxygenases

**DOI:** 10.1016/j.jbc.2025.108241

**Published:** 2025-01-27

**Authors:** Inchul Shin, Romie C. Nguyen, Samuel R. Montoya, Aimin Liu

**Affiliations:** Department of Chemistry, The University of Texas at San Antonio, Texas, United States

**Keywords:** heme, oxygenation mechanism, HDAO superfamily, monooxygenation, crystal structure

## Abstract

MarE, a heme-dependent enzyme, catalyzes a unique 2-oxindole-forming monooxygenation reaction from tryptophan metabolites. To elucidate its enzyme-substrate interaction mode, we present the first X-ray crystal structures of MarE in complex with its prime substrate, (2*S*,3*S*)-**β**-methyl-l-tryptophan and cyanide at 1.89 Å resolution as well as a truncated yet catalytically active version in complex with the substrate at 2.45 Å resolution. These structures establish MarE as a member of the heme-dependent aromatic oxygenase (HDAO) superfamily and reveal its evolutionary link to indoleamine 2,3-dioxygenase (IDO) and tryptophan 2,3-dioxygenase (TDO). While MarE adopts a global structure resembling the homotetrameric TDO, it features a simplified **α**6 helix compared to TDO’s more elaborate **α**E and **α**H helices with additional **α**F and **α**G regions. Despite differing oxygen activation outcomes, MarE shares a substrate binding mode similar to IDO and TDO, with the indole nitrogen of its substrate oriented toward the heme iron in the ternary cyano complex, interacting with His55. The substrate’s carboxylate group engages Arg118, with mutational studies confirming the roles of these residues in substrate binding. However, the second-sphere interactions with the substrate’s **α**-amino nitrogen differ between MarE and TDO, and the substrate’s orientation in the binary complex remains ambiguous due to two possible conformations. Notably, TDO features an extensive hydrogen-bonding network around the heme propionate below the heme plane, which is absent in MarE, suggesting mechanistic differences. These structural insights lay a foundation for further mechanistic studies, particularly for understanding how heme-dependent enzymes oxygenate tryptophan-derived metabolites.

MarE is a bacterial enzyme that catalyzes a unique 2-oxindole production reaction through monooxygenation of (2*S*,3*S*)-β-methyl-l-tryptophan (β-Me-l-Trp) (Fig. 1) (1). The 2-oxindole product serves as a critical scaffold for the synthesis of bioactive natural products known as maremycins, which exhibit antimicrobial activity (1, 2).

**Figure 1:**
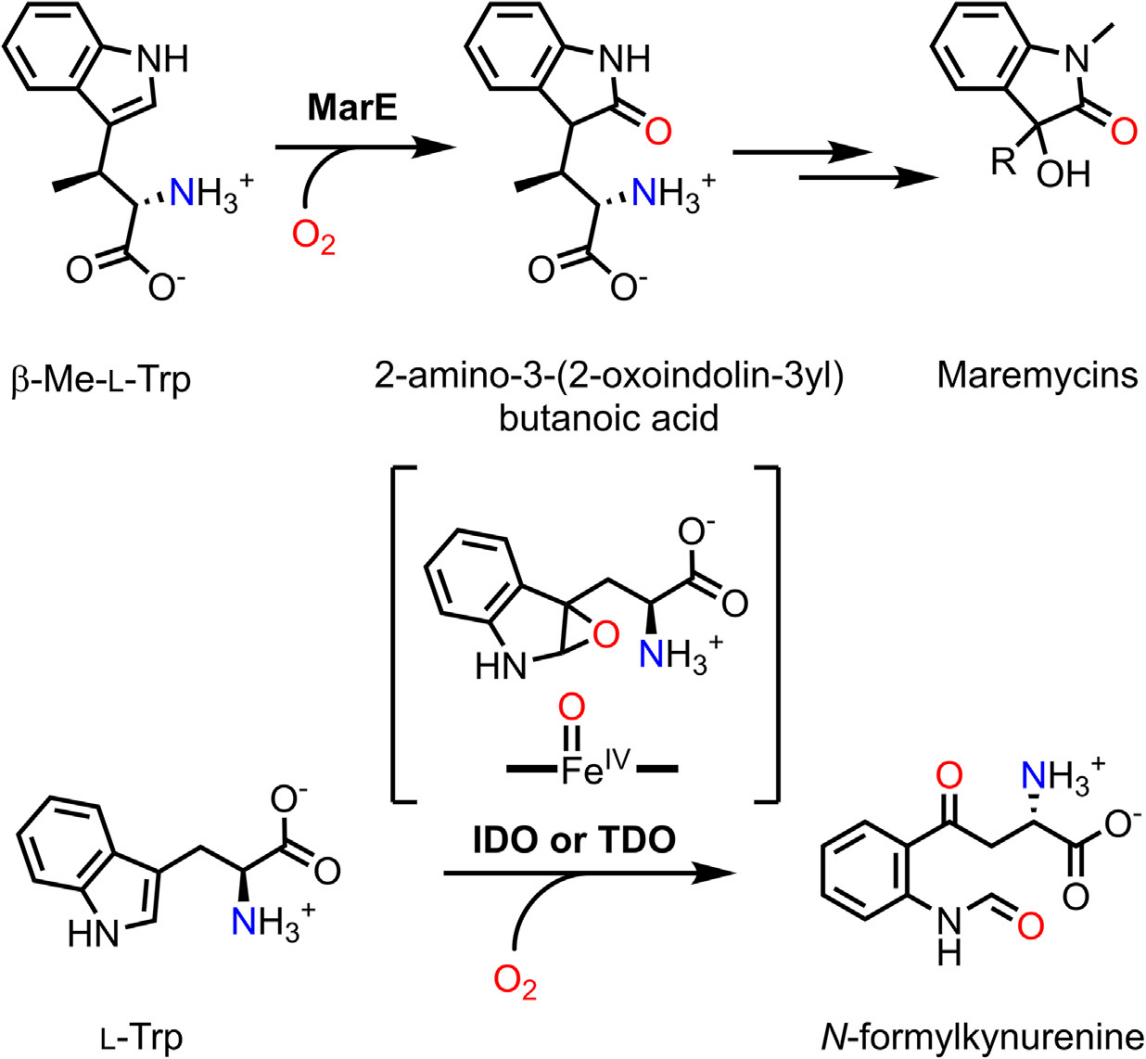
**Chemical reactions catalyzed by MarE (*top*) and IDO or TDO (*bottom*)**.

The amino acid sequence of MarE shares significant similarity with tryptophan 2,3-dioxygenase (TDO) (1) (Fig. 2), a histidine-ligated heme-dependent enzyme that catalyzes the activation and insertion of molecular oxygen by a histidine-ligated heme into the indole ring of l-tryptophan (l-Trp), producing *N*-formylkynurenine (NFK) (3, 4, 5, 6). TDO and its closely related homolog, indoleamine 2,3-dioxygenase (IDO), catalyze stepwise O-atom transfers, completing dioxygenation through two sequential monooxygenation reactions (7, 8, 9, 10). However, MarE diverges functionally by catalyzing only a single monooxygenation reaction on a tryptophan metabolite, terminating the oxidation process before full dioxygenation (1).

**Figure 2:**
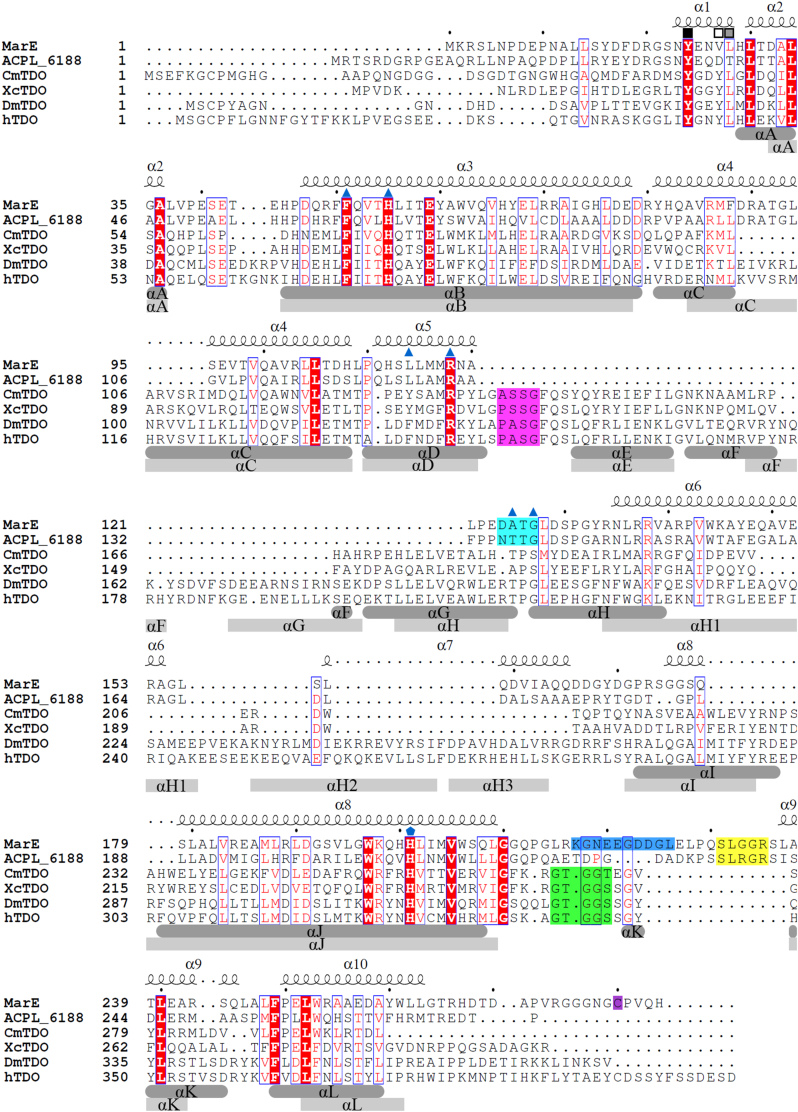
**Sequence alignments of MarE.** Secondary structures of MarE are labeled on the top of the sequences. Secondary structures of XcTDO (PDB entry: 2NW8) with a *dark gray round rectangle* and hTDO (PDB entry: 5TIA) with a *light gray rectangle* are labeled at the *bottom* of the sequences. Squares represent the residues originating from the neighboring subunit. The closed black square is for the strictly conserved Tyr∗. *Gray* and *open squares* are for the conserved and not-conserved residues. *Blue triangles* are key residues in the heme active sites. Asp124, Ala125, Thr126, and Gly127 (DATG) of MarE and the corresponding region, Pro149, Ala150, Ser151, and Gly152 (PASG) in hTDO are *shaded* with *cyan* and *magenta*, respectively. Heme axial ligand is marked with a *blue pentagon*. SLGGR in MarE and GTGGS in TDOs are *shaded yellow* and *green*, respectively. The disordered region is *shaded* with *blue*. The C-terminal Cys280 is *shaded purple*.

Recent bioinformatic and structural analyses have suggested that these tryptophan dioxygenase enzymes are part of a larger protein group known as the heme-dependent aromatic oxygenase (HDAO) superfamily, which is expanded from the previously recognized TDO superfamily (11). The HDAO superfamily represents a structurally based and functionally related group of proteins with a histidyl-ligated heme for primarily oxygenation activities. It rivals the well-established heme-based superfamilies such as cytochromes P450 and peroxygenases with cysteine-ligated heme driving the reaction. Beyond catalyzing the dioxygenation reaction on tryptophan, these structurally related superfamily members exhibit functional diversity, including monooxygenation and possibly coupled monooxygenation and decarboxylation (1, 11, 12, 13, 14, 15, 16). Moreover, the superfamily's substrate repertoire has expanded beyond tryptophan and its derivatives, including tyrosine and tyrosine-derived metabolites (11, 12, 13, 14, 17).

Our bioinformatic analysis indicates that MarE may represent a new subgroup of enzymes to the HDAO superfamily, uniquely positioned in the phylogenetic tree at the boundary between the TDO subgroup and a tyrosine metabolite monooxygenase subgroup, represented by the characterized member SfmD (11). However, the definitive classification of MarE within this protein family requires structural validation. The structural details of heme-bound holo MarE remain unavailable, and the lack of substrate-bound structures impedes mechanistic investigations.

To address these gaps, we have determined the three-dimensional structure of MarE. Herein, we report the crystal structures of MarE in complex with its native substrate β-Me-l-Trp and compare the structural features and enzyme-substrate interaction mode with TDO.

## Results

### Crystallization of MarE

Achieving MarE crystallization required overcoming two significant hurdles. The first challenge was the low heme occupancy observed in the isolated protein. Bacterial cell cultures supplemented with δ-aminolevulinic acid (heme precursor) and iron (II) ion resulted in a maximum heme occupancy of only 20%. Fortunately, we found that heme reconstitution was possible for MarE, which increased the heme occupancy to over 70%, as confirmed by the pyridine hemochromagen method (Fig. S1*A*) (18).

The other obstacle encountered during crystallization was the heterogeneity observed in the gel-filtration chromatography elution pattern, which showed multiple peaks rather than a single well-defined peak (Fig. S1*B*). Further investigation revealed that Cys280 at the C-terminus was reactive, forming crosslinks and causing heterogeneity. To address this, we generated C280S variant and Δ(270–284), a C-terminal truncated version using PCR with primers listed in the Experimental procedures section. The untagged variant displayed a monodispersed gel-filtration chromatography pattern (Fig. S1*B*) and was subsequently utilized for crystallization experiments.

**Figure 3:**
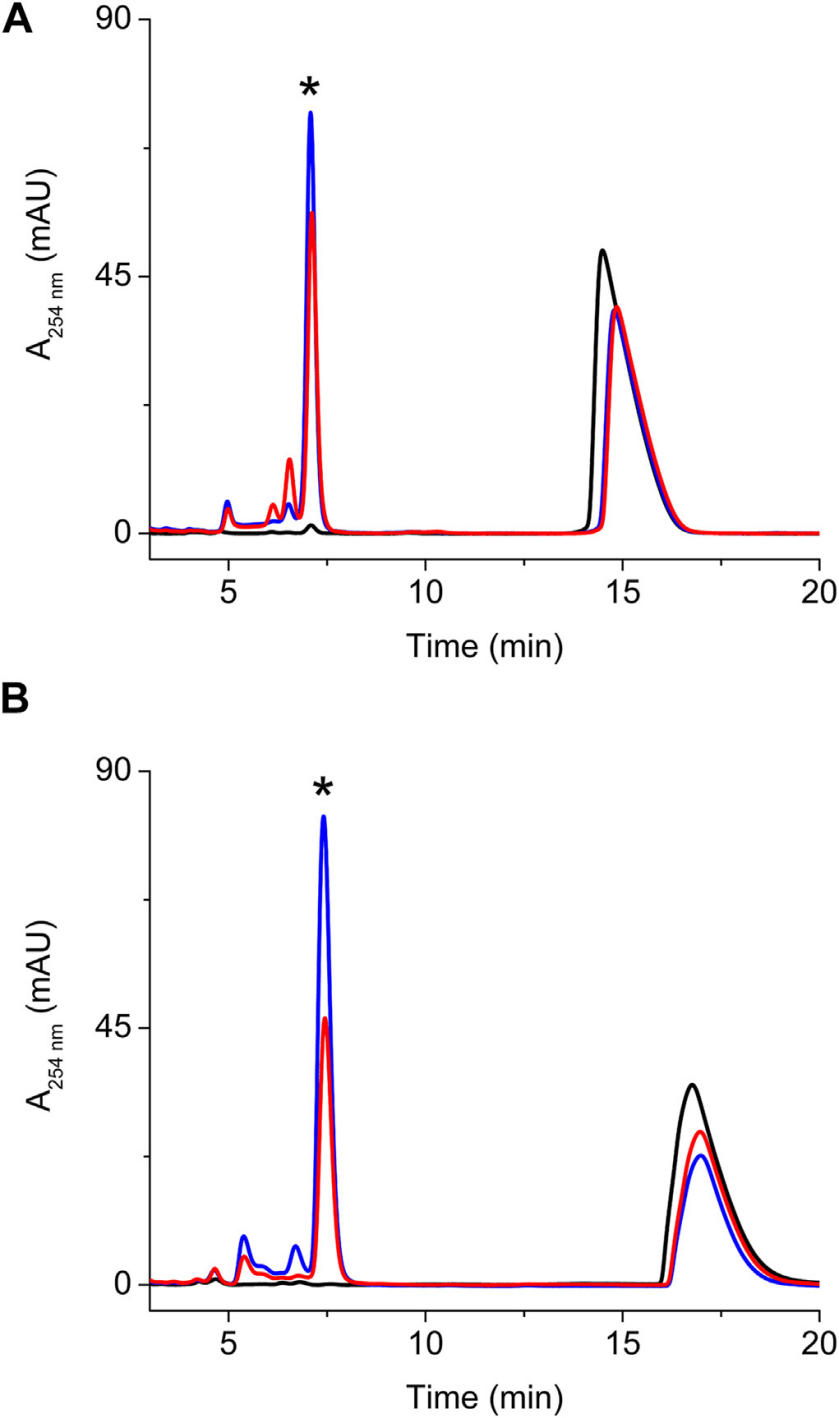
**MarE C280S and MarE Δ(270–284) are catalytically competent**. *A*, β-Me-l-Trp (*black trace*), wild-type MarE + β-Me-l-Trp + ascorbate (*blue trace*), and MarE C280S + β-Me-l-Trp + ascorbate (*red trace*). *B*, β-Me-l-Trp (*black trace*), wild-type MarE + β-Me-l-Trp + ascorbate (*blue trace*), and MarE Δ(270–284) + β-Me-l-Trp + ascorbate (*red trace*). The reactions were carried out aerobically and monitored by HPLC. The concentrations for β-Me-l-Trp, enzyme, and ascorbate were 1 mM, 50 μM (heme) and 20 mM, respectively. The elution peak labeled with an *asterisk* mark (∗) indicates the monooxygenated 2-oxindole product of β-Me-l-Trp.

The heme-reconstituted MarE protein was catalytically active. MarE C280S and MarE Δ(270–284) also retained catalytic activity, although less efficient than full-length wild-type MarE (Fig. 3). To achieve these assays, we enzymatically synthesized β-Me-l-Trp using an engineered tryptophan synthase (Figs. S2 and S3) (19).

MarE C280S crystals were obtained by crystallization in the presence of 50 mM sodium cyanide and 10 mM β-Me-l-Trp. MarE Δ(270–284) was crystallized in the presence of 10 mM β-Me-l-Trp but without the inclusion of sodium cyanide. The co-crystalized structures were determined at 1.89 Å and 2.45 Å resolutions using molecular replacement, with an AlphaFold-predicted structure serving as the initial search model for phasing assistance (Table 1) (20). While AlphaFold typically provides highly accurate predicted structures, its present form lacks critical information, such as enzyme cofactor binding and substrate-induced structural changes.

**Table 1:** X-ray diffraction data collection and refinement statistics

	Ternary complex	Binary complex
Data collection		
Space group	*P*2_1_	*I*422
Cell dimension		
a, b, c (Å)	71.03, 112.96, 83.34	196.12, 196.12, 113.06
α, β, γ (°)	90.00, 111.25, 90.00	90.00, 90.00, 90.00
Resolution (Å)	50.00–1.89 (1.92–1.89)^a^	50.00–2.45 (2.58–2.45)
Total reflections	612,990	1,010,353
Unique reflections	94,295	40,492
*R*_merge_ (%)	16.0 (108.9)	17.0 (234.8)
I/σI	12.55 (1.21)	21.2 (1.0)
Completeness (%)	96.6 (85.2)	100 (99.9)
CC_1/2_	0.977 (0.670)	0.999 (0.385)
Refinement		
Resolution (Å)	43.24–1.89	43.85–2.45
No. of reflections	94,052	39,790
*R*_work_/*R*_free_ (%)	19.33/24.09	17.66/20.95
No. atoms/*B*-factor (Å^2^)		
Protein^b^	8234/39.1	3941/56.7
Heme	172/36.4	86/51.3
Cyanide	8/33.4	N/A
β-Me-l-Trp	64/38.7	48/60.6
Water	520/42.0	92/54.8
RMSD		
Bond length (Å)	0.009	0.008
Bond angle (°)	1.106	1.148
Ramachandran statistics		
Favored (%)	98.62	95.83
Allowed (%)	1.28	3.34
Outlier^c^ (%)	0.10	0.83
PDB code	9CA3	8VYY

a Values in parentheses are for the highest resolution shell.

b Ordered residues: For ternary complex, A/Met1–Arg216 and Glu227–Thr265; B/Lys2–Leu215 and Leu228–Val273; C/Lys2–Arg216 and Leu228–Pro272; D/Met1–Leu215 and Glu227–Thr269. For A/Glu44, A/Glu123, and D/Arg235, sidechains were omitted due to a lack of electron densities. For binary complex, A/Ser4– Arg216 and Arg235–Thr265; B/Ser4–Arg216 and Arg235–Thr265.

c Ramachandran outlier: For ternary complex, D/Pro10. For binary complex, A/Leu5, A/Glu9, B/Glu9, and B/Pro122.

### Overall structure of MarE

**Figure 4:**
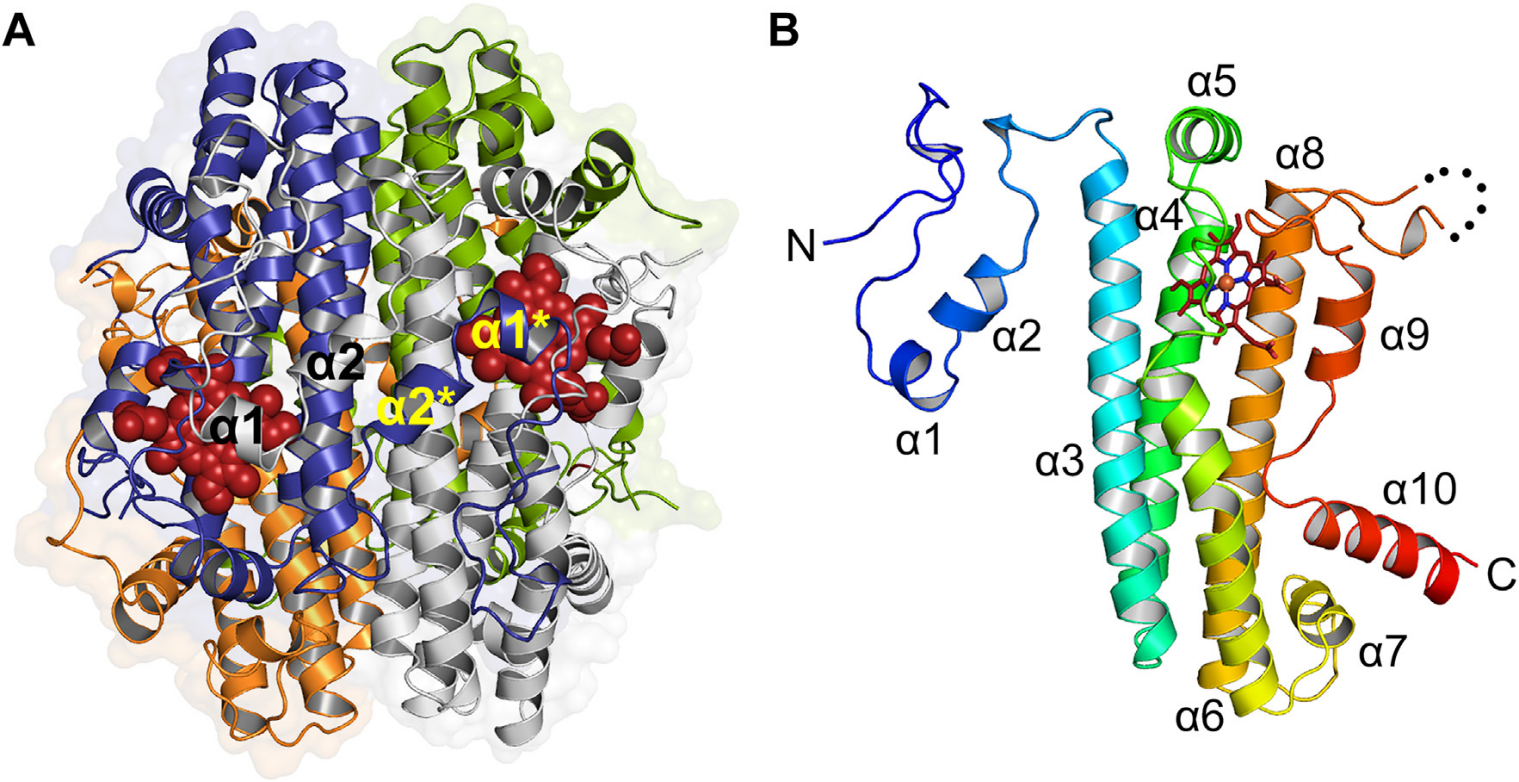
**Crystal structure of MarE**. *A*, asymmetric unit contains four subunits of MarE monomer *colored* in *gray*, *dark blue*, *orange*, and *green*. Heme is presented in the *red sphere* model. *B*, a side view of a monomeric subunit in *rainbow color* scale from *blue* to *red*, denoting N- to C- termini. Residues ranging from 217 to 226 are disordered between helices α8 and α9 and are shown in *black dots*. Heme is presented in a *red stick* model.

**Figure 5:**
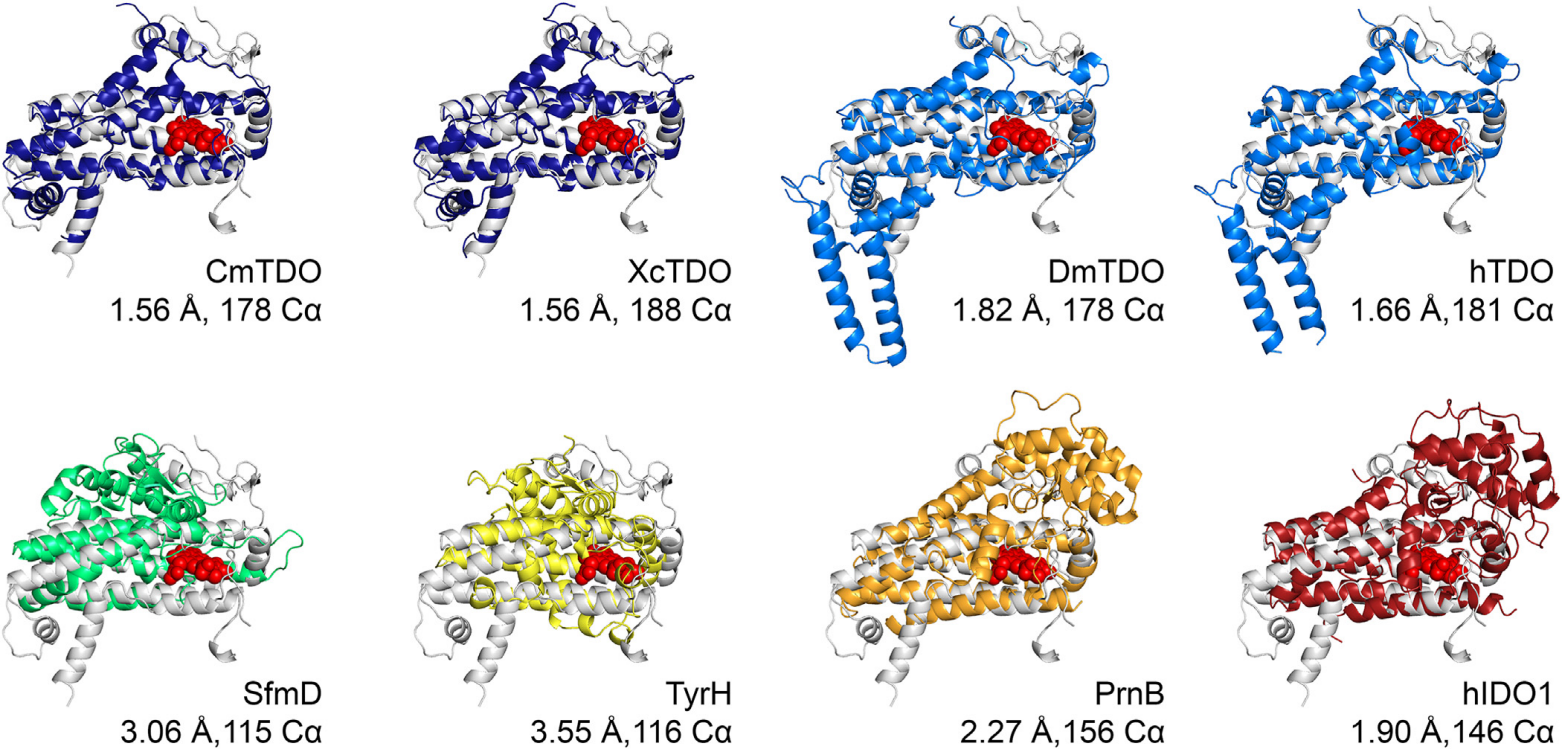
**Superposition of MarE on the structurally known members of the HDAO superfamily establishes that MarE is a structurally verified member of the HDAO superfamily**. Heme is presented in the *red sphere* model.

The MarE C280S crystal belongs to a primitive monoclinic space group of *P*2_1_ and contains four protomers in an asymmetric unit (Fig. 4*A*). The structure determined at 1.89-Å resolution forms a dimer of dimers, consistent with the homo-tetrameric oligomeric state of MarE in the solution state (Fig. 3*B*), without requiring further crystallographic symmetry operations. Each monomer consists of ten α-helices, with a disordered region spanning residues of 217 to 226 (Figs. 2 and 4*B*). Helices α1 and α2 protrude towards the neighboring subunit, interlocking to stabilize the dimer (Fig. 4*A*). Helices α3, α4, α6, and α8 form a four-helix bundle core, with α5 acting as a lid (Fig. 4*B*). Notably, this core helix bundle with a lid is a consensus structural element found across all characterized members of the HDAO superfamily (Fig. 5). MarE structurally aligns with TDO structures from various sources (21, 22, 23, 24), with root-mean-square deviation (RMSD) values ranging from 1.56 to 1.82 Å for 178 to 188 Cα atoms (Fig. 5). This structural analysis places MarE into the HADO superfamily as the newest structurally validated member.

A noticeable overall structural difference between MarE and TDOs is the absence of helices αF and αG, which are present in human TDO (hTDO) and the well-characterized bacterial TDOs from *Cupriavidus metallidurans* (CmTDO) and *Xantomonas campestris* (XcTDO) (Figs. 2 and S4) (18, 21). Instead, MarE features a simplified single helix, α6, corresponding to the combined helices αE and αH of TDOs (Fig. 4*B*).

### Active site architecture and the binding mode of **β**-Me-l-Trp in the ternary complex structure

At the enzyme active site, the heme is located within the core helix of MarE, near the lid helix α5, with well-defined electron density (Figs. 4 and S5). Four heme groups are found in the structure, one each per polypeptide. Although the heme content was increased through the heme-reconstitution reaction, the heme was positioned identically in each subunit. We noticed that the uniform orientation of the heme bound is mainly due to Thr58 (Fig. S5). When flipping the heme, the 4-vinyl group of the heme clashes with Thr58.

**Figure 6:**
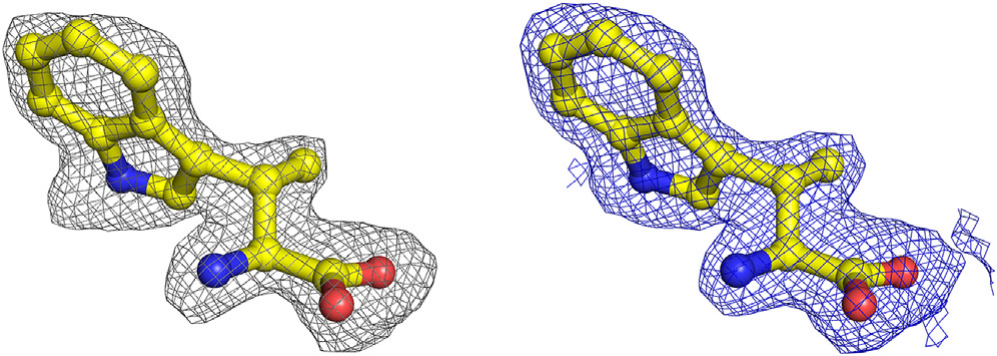
**Electron density maps for β-Me-l-Trp bound in the active site of MarE**. A representative omit *F*_o_–*F*_c_ map contoured at 3 σ, and 2*F*_o_–*F*_c_ map contoured at 1 σ are shown in *gray* and *blue*, respectively. β-Me-l-Trp is presented in *yellow carbon*.

We identified electron density above the heme iron, into which we modeled cyanide (Figs. S5 and S6). Additionally, we observed extra electron density above the cyanide-bound heme, where we modeled β-Me-l-Trp (Figs. 6 and S7). The indole ring and carboxylate were placed as head and tail, allowing unambiguous assignment of the amino and β-methyl groups into a single conformation. In this conformation, the substrate amino group points toward the heme iron center, similar to the substrate binding mode observed in the binary enzyme-substrate complex of IDO/TDO (21, 24, 25). We also explored an alternative binding mode, where the amino and β-methyl groups of β-Me-l-Trp were flipped. However, this conformation resulted in poor geometry and fitting against the experimental density, leading to negative *F*_o_–*F*_c_ signals and clashes with surrounding residues (Fig. S8).

**Figure 7:**
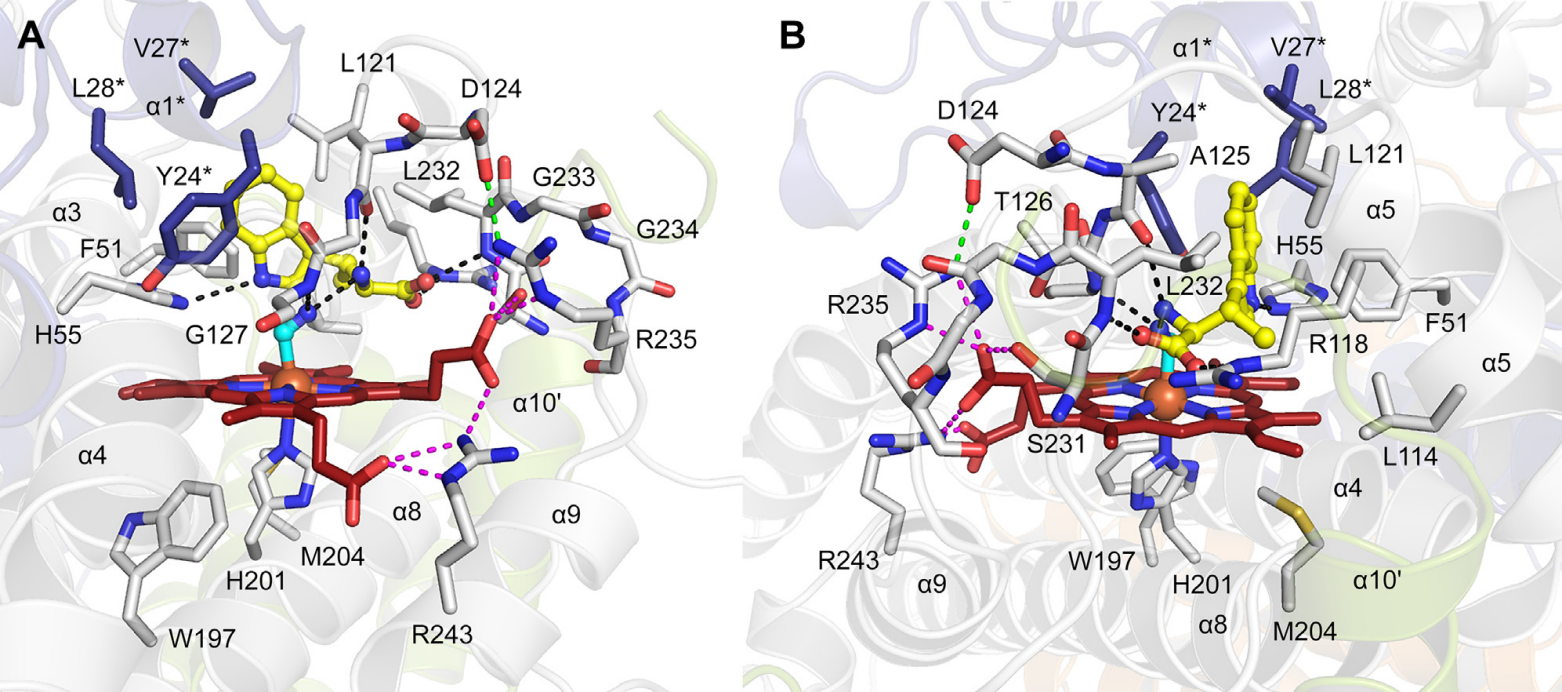
**Cyanide and β-Me-l-Trp bound active site structure of MarE**. The active site is shown in two different orientations in *A* and *B*. Heme, cyanide, and β-Me-l-Trp are presented in *red*, *cyan*, and *yellow carbon color*, respectively. Residues from neighboring subunits are presented in *deep blue* with the label ∗. Helix α10ʹ is from another neighboring subunit *colored* in *light green*. *Dotted lines* denote interaction within a 3.2 Å distance.

His201 serves as the axial ligand anchored to the heme and contacts with Trp197 and Met204 further stabilize the prosthetic group (Fig. 7). The two heme propionates adopt distinct conformations relative to the heme plane: heme propionate-6 is positioned above the heme plane, interacting with Ser231, Arg235 of the SLGGR loop, and Arg243, while heme propionate-7 is laid below the heme plane and interacts solely with Arg243.

MarE active site is organized into three key regions: a deep hydrophobic pocket located at the innermost part of the active site, the lid helix α5 and its adjacent loop region (Asp124-Ala125-Thr126-Gly127, DATG), and an ordered loop region between α8 and α9 (Ser231-Leu232-Gly-233-Gly234-Arg235, SLGGR) near the bulk solvent, which shields the active site (Fig. 7). Residues from the neighboring subunit, including Tyr24∗, Val27∗, and Leu28∗, along with Phe51 and Leu121, create a deep hydrophobic pocket that accommodates the indole ring of β-Me-l-Trp. His55 is positioned beneath Leu28∗ and, analogous to His72 in CmTDO, a known catalytic facilitator (26), forms an H-bond with the indole nitrogen atom of the bound β-Me-l-Trp.

The β-methyl group of the substrate is directed toward a hydrophobic pocket formed by Phe51, Leu114, and Leu121. The substrate amino group is oriented towards the heme center, interacting with the main-chain carbonyl oxygen of Ala125 and the nitrogen of cyanide. The cyanide nitrogen, in turn, interacts with the main-chain nitrogen of Gly127, both of which are part of the DATG loop originating from the lid helix α5 and extending through the active site. The carboxylate group of the substrate interacts with Nε of Arg118 from the lid helix α5 and the main-chain nitrogen of Leu232, a component of the SLGGR loop.

### Mutational assessment of the critical second sphere residues

To evaluate the physiological relevance of the observed substrate-binding mode through crystallization, site-directed mutagenesis was performed at residues His55 and Arg118, both implicated in substrate recognition. Mutations were introduced into the C280S variant to ensure an unbiased comparison during crystallographic studies.

**Figure 8:**
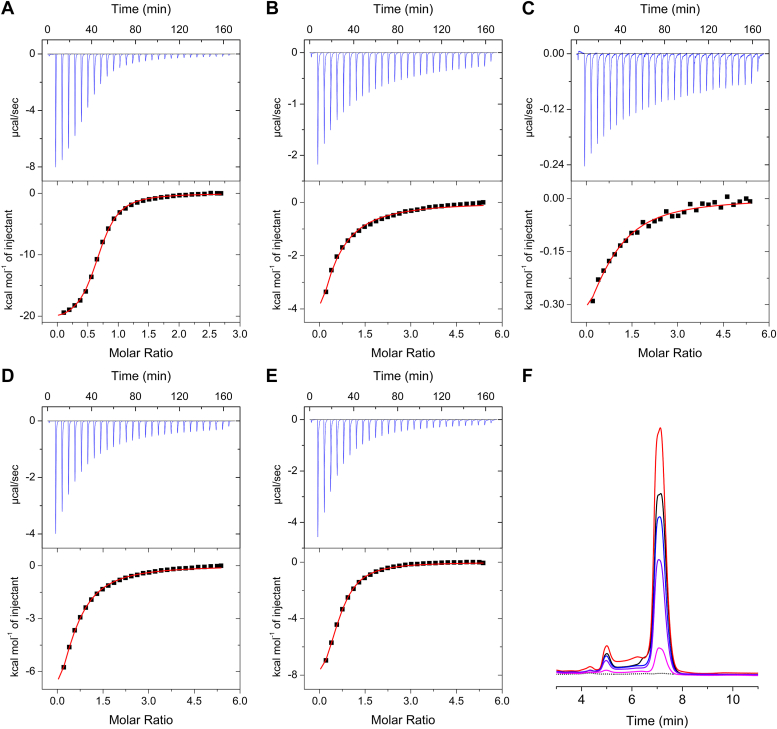
**Mutations at His55 and Arg118 significantly reduced substrate affinity**. β-Me-l-Trp binding was measured by ITC. Representative data is presented among the triplicate measurements. *A*, C280S. *B*, C280S/H55A. *C*, C280S/H55F. *D*, C280S/R118A. *E*, C280S/R118K. *F*, the product formation was analyzed through HPLC-based analysis. C280S (*black trace*), C280S/H55A (*red*), C280S/H55F (*magenta*), C280S/R118K (*blue*), C280S/R118A (*purple*), and β-Me-l-Trp (*black dotted*).

The dissociation constants (*K*_D_) values for β-Me-l-Trp binding to C280S, C280S/H55A, C280S/H55F, C280S/R118A, and C280S/R118K were determined using isothermal titration calorimetry (ITC), yielding values of 4.57 ± 0.20 μM, 92.6 ± 6.4 μM, 98.6 ± 9.5 μM, 53.6 ± 2.3 μM, and 23.7 ± 1.1 μM, respectively (Fig. 8 and Table 2). Mutations at both positions significantly reduced substrate affinity, with mutations at His55 exhibiting a greater impact than those at Arg118. Among the Arg118 mutations, the conserved substitution R118K resulted in the smallest increase in *K*_D_, with an observable reduction in affinity.

**Table 2:** ITC parameters

	N	*K*_D_ (μM)	ΔH (cal/mol)	ΔS (cal/mol/deg)
C280S	0.66 ± 0.00	4.57 ± 0.20	−2.088 × 10^4^ ± 320	−46.3 ± 1.1
C280S/H55A	0.27 ± 0.04	92.6 ± 6.4	−1.705 × 10^4^ ± 2672	−39.3 ± 9.2
C280S/H55F	0.64 ± 0.09	98.6 ± 9.5	−0.772 × 10^4^ ± 127	15.7 ± 0.6
C280S/R118A	0.50 ± 0.00	53.6 ± 2.3	−1.376 × 10^4^ ± 415	−27.1 ± 1.5
C280S/R118K	0.59 ± 0.01	23.7 ± 1.1	−1.079 × 10^4^ ± 146	−15.4 ± 0.6

Product formation assays using HPLC revealed a general decrease in product yield for all mutants compared to C280S, aligning with the observed reductions in binding affinity (Fig. 8*F*). However, the C280S/H55A mutant showed an unexpected increase in product formation despite a ∼20-fold decrease in substrate affinity. This finding suggests that the H55A mutation may have altered the enzyme’s conformational dynamics or substrate orientation, leading to enhanced catalytic efficiency. Given that MarE functions in secondary metabolism, its catalytic efficiency may be naturally tuned to operate at a slower rate, potentially to synchronize with other steps in the maremycin biosynthetic pathway. Thus, it is not surprising for a mutant to exhibit an improved catalytic rate.

### Structural comparison between MarE and TDO

**Figure 9:**
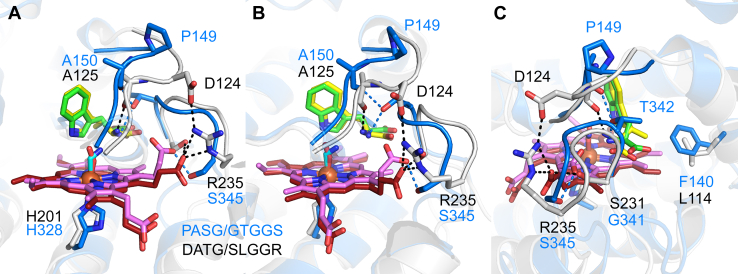
**Active site comparison with hTDO.** The superposed active sites of hTDO and MarE are shown in three different orientations, *A*–*C*, protein residues and heme of hTDO are presented in *blue* and *pink carbon color*. Protein residues and heme of MarE are shown in *gray* and *red colors*. l-Trp and β-Me-l-Trp are shown in *green* and *yellow carbon color*. *Dotted lines* denote interaction within a 3.2 Å distance.

MarE exhibits a simplified helix α6, corresponding to a combination of the αE and αH of TDOs, which possess additional secondary structural elements, αF and αG. Superposition of the oxygen-bound hTDO structure (PDB entry: 5TI9) onto MarE revealed two notable structural differences in the active site (Fig. 9).

First, the loop DATG in MarE is distinct from the corresponding PASG loop in hTDO. In MarE, DATG is positioned closer to the solvent front due to an interaction between Asp124 of DATG and Arg235 of SLGGR. This conformation allows the main chain carbonyl oxygen of Ala125 in DATG to form a hydrogen bond with the amino group of β-Me-l-Trp. In contrast, in hTDO, the amino group of l-Trp is 4.6 Å away from the equivalent carbonyl oxygen of Ala150 in PASG, instead interacting with Thr342 of the GTGGS loop. Thr342, in turn, interacts with the main chain carbonyl oxygen of Ala150 in PASG (Fig. 9).

**Figure 10:**
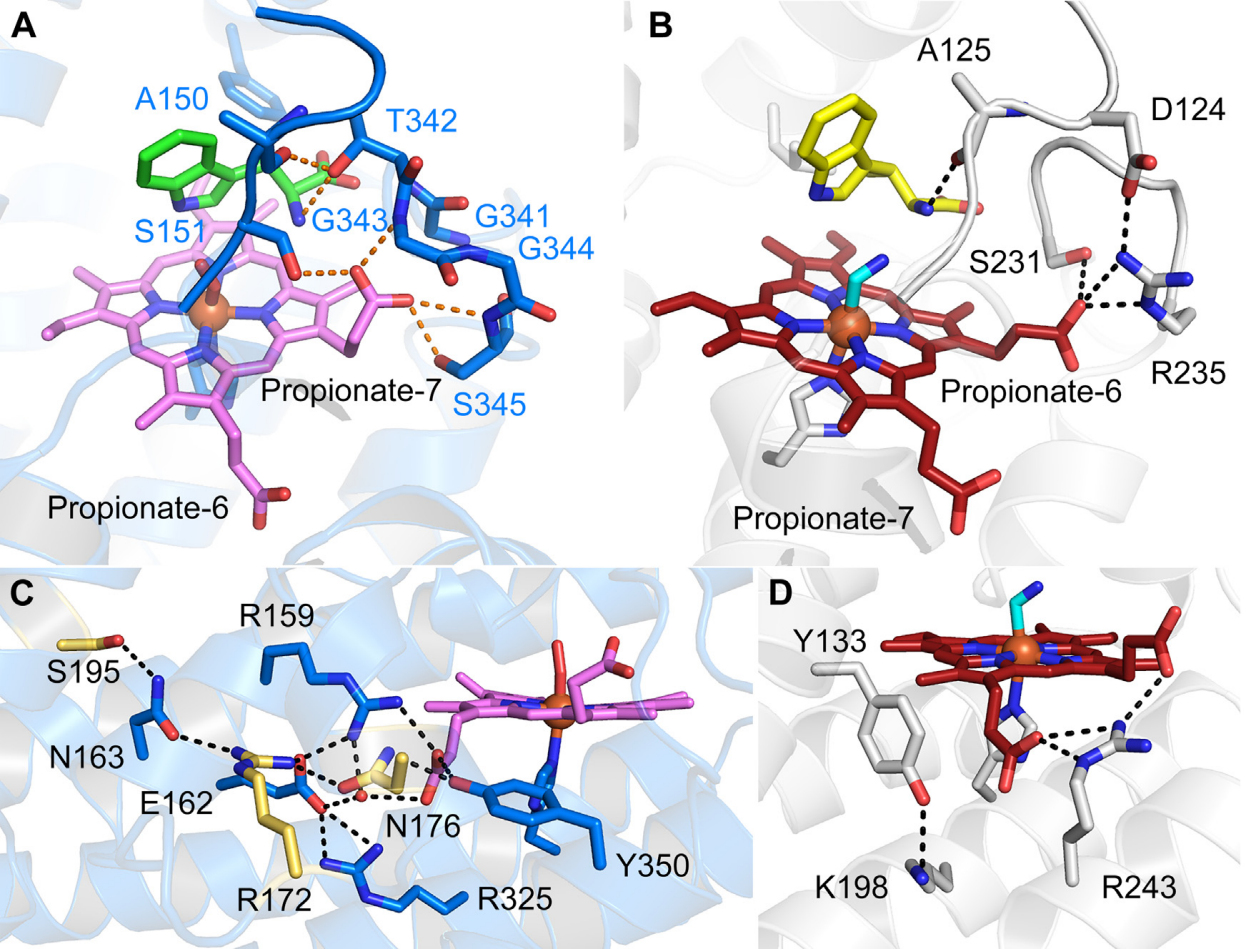
**Differences in heme propionates environment in hTDO and MarE**. *A*, hydrogen-bonding network established between PASG/GTGGS and heme propionate-7 in hTDO. *B*, interaction between DATG/SLGGR and heme propionate-6 in MarE. *C*, extensive hydrogen bonding network starting from heme propionate-6 in hTDO. *D*, The environment near heme propionate-7 in MarE is simpler than hTDO. Protein residues and heme of hTDO are presented in *blue* and *pink carbon color*. *Golden-colored* residues stem from extra helices, αF and αG. Protein residues and heme of MarE are shown in *gray* and *red carbon color*. l-Trp and β-Me-l-Trp are shown in *green* and *yellow carbon color*. *Dotted lines* denote interaction within a 3.2 Å distance.

Second, the loop SLGGR in MarE superposes the GTGGS loop in hTDO, with an RMSD of 0.11 Å for 5 Cα atoms, indicating nearly identical widths (Fig. S9). The GTGGS loop in hTDO interacts with the heme propionate-7 *via* the main chain nitrogen atoms of Gly343 and Ser345, as well as Ser345 itself (Fig. 9). Notably, in hTDO, heme propionate-7 is positioned above the heme plane, reflecting a flipped heme orientation. This propionate forms a hydrogen bond with Ser151, bridging the PASG and GTGGS loops in hTDO (Fig. 10*A*). In contrast, the SLGGR loop in MarE is tilted, preventing a similar interaction. The heme propionate-6 in MarE adopts a stretched conformation, interacting only with Ser231 and Arg235 of SLGGR, without engaging the DATG loop (Fig. 10*B*).

A further difference between MarE and hTDO involves the heme propionate below the plane. In hTDO, heme propionate-6 participates in an extensive hydrogen-bonding network with Arg172, Asn176, and Ser195, residues stemming from the extra secondary structural elements, αF and αG (Fig. 10*C*). In contrast, MarE’s heme propionate-7 interacts solely with Arg243, with further hydrogen bond formation seemingly blocked by Tyr133 and Lys198 (Fig. 10*D*).

### Binary enzyme-substrate (ES) complex structure

Co-crystallizing the binary enzyme-substrate (ES) complex structure proved challenging, likely due to the substrate not being directly ligated to the heme and its slow reduction of ferric heme when cyanide ligation is absent, similar to TDO (27). Unlike the successful crystallization of the full-length protein in the ternary complex, we resolved an ES crystal structure of truncated MarE Δ(270–284) at 2.45 Å resolution through co-crystallization with β-Me-l-Trp. Unlike the ternary crystal, the MarE Δ(270–284) crystal belongs to a body-centered tetragonal space group (*I*422) and contains two protomers in the asymmetric unit (Fig. S10). The dimer-of-dimer structure was constructed through crystallographic symmetry operations, consistent with MarE's homotetrameric oligomeric state in solution (Fig. S11). Each monomer structure is nearly identical to the ternary cyano complex, with an RMSD of 0.54 Å for 243 Cα atoms, though it features a longer disordered region spanning residues 217 to 234 (Fig. S12).

In the ES complex structure, we identified electron densities corresponding to the heme (Fig. S13) and modeled β-Me-l-Trp into this additional density (Fig. S14). In chain A, two substrate-binding modes were observed at the heme distal pocket. One conformation (conformation A) is identical to that seen in the ternary cyano complex (Fig. S14*A*, substrate with yellow carbons). The other (conformation B) is a new orientation where the substrate's amino nitrogen points away from the heme center, while the methyl group points toward it (Fig. S14*A*, substrate with cyan carbons).

Interestingly, chain B exclusively displays conformation B in the ES complex structure of truncated MarE Δ(270–284) (Fig. S14*B*). Attempts to fit conformation A into the observed electron density were unsuccessful, as the substrate did not align with the 2*F*_o_–*F*_c_ map during refinement (Fig. S15). Substrate occupancies were adjusted until no negative *F*_o_–*F*_c_ signals remained after refinement: for chain A, 0.10 for conformation A, 0.56 for conformation B, yielding an overall occupancy of 0.66; and for chain B, 0.75 for conformation B.

In the binary complex structure, the SLGGR loop is disordered, consistent with the disordered region spanning residues 217 to 234. The position of Arg235, marking the start of the ordered region in the binary complex, differs significantly from its location in the ternary complex (Fig. S16*A*). While the ternary complex also exhibits a disordered region (residues 217–226), the SLGGR loop becomes ordered starting from Glu227, contributing a crucial structural element to the heme active site (Fig. S16). As a result of the disordered SLGGR loop in the binary complex, Asp124 of the DATG loop remains in a retracted position, no longer interacting with Arg235 of SLGGR. Additionally, the heme propionate-6 in the binary complex curls back, severing its interaction with Arg235 (Fig. S16).

## Discussion

### Structural insights into MarE: challenges and strategies for crystallization

MarE has garnered attention for two main reasons: first, oxindole serves as the core structure of an expanding class of natural products, such as maremycins, known for their biological activities (28, 29, 30, 31, 32, 33, 34, 35, 36, 37, 38, 39, 40). Second, as an oxindole-installing monooxygenase, MarE is closely related—both in sequence and function—to the biomedically significant tryptophan dioxygenases IDO and TDO. consequently, we initiated an investigation into its protein scaffold and enzyme-substrate interaction mode as a foundational step toward understanding the factors that govern monooxygenation *versus* dioxygenation of the tryptophan indole ring. However, achieving homogeneity in MarE protein crystallization posed a significant challenge. To address this, we introduced a surface cysteine mutation, a strategy previously shown to be effective for human cysteamine dioxygenase facing similar difficulties (41).

### Structural divergence between MarE and TDO in the HDAO superfamily

The structural advance presented in this work unambiguously shows that MarE shares a close structural relationship with TDO within the HDAO superfamily. However, we identified significant conformational differences in the active site loops: DATG/SLGGR in MarE and PASG/GTGGS in hTDO. These differences lead to distinct hydrogen bonding interactions with the substrate amino group. In MarE, the substrate amino group forms an H-bond with the mainchain carbonyl oxygen of Ala125. In contrast, in hTDO, the amino group of l-Trp interacts with the hydroxyl group of Thr342. The nature of these H-bond partners—one being a carbonyl oxygen, the other a hydroxyl group—could be critical in driving the second O-atom transfer step by assisting with the epoxide ring opening.

The loop conformation differences also influence the positioning of heme propionates above the heme plane. In MarE, heme propionate-6 extends outward, interacting with Ser231 and Arg235 of the SLGGR loop. The DATG and SLGGR loops are joined by a key interaction between Asp124 and Arg235. Conversely, in hTDO, heme propionate-7 is rolled up, mediating the interaction between the PASG and GTGGS loops, along with a hydrogen bond between the mainchain carbonyl oxygen of Ala150 and Thr342.

### Impact of loop conformation on substrate dynamics

The GTGGS loop in hTDO plays a crucial role in substrate loading, product release, and active site sequestration from bulk solvent (42, 43). In MarE, the SLGGR loop forms a relatively loose connection to the DATG loop. This less tightly controlled loop opening and closure might contribute to the failure in the second O-atom transfer, potentially leading to monooxygenation due to the reduction of compound II by reducing agents such as ascorbate.

### Substrate conformation and binding mode

Intriguingly, the β-Me-l-Trp in the binary complex exhibited mixed binding modes, with conformation B, where the amino nitrogen is positioned away from the iron, being the more prevalent. In substrate-bound TDO structures, the presence of a protein loop structure, *i.e.*, the JK-loop, restricts the rotation of the amino group, enforcing an orientation similar to conformation A, with the amino nitrogen positioned toward the iron-bound ligand during catalysis. In contrast, the corresponding JK-loop in the binary structure of MarE is disordered (Fig. S16). This disordered JK-loop likely contributes to the emergence of two substrate-binding modes in the MarE binary complex. However, two complicating factors should be considered. First, the low resolution of the structure introduces some ambiguity. Second, the C-terminus of MarE, including Cys280, is disordered. The C-terminus is adjacent to the active site of a neighboring subunit, and its absence may introduce additional substrate binding flexibility or dynamics because mutation and truncation in this region reduce the catalytic efficiency (Fig. 3).

Conversely, in the 1.89-Å ternary complex structure with full-length (1–284) MarE C280S, the β-Me-l-Trp is unambiguously assigned, with the substrate amino group oriented toward the heme center and the β-methyl group directed toward the hydrophobic pocket in all four subunits. In the dioxygenation reaction catalyzed by IDO/TDO, the substrate amino group is proposed to play a crucial role in the second oxygen atom transfer step, assisting in the epoxide ring opening (7, 8, 9, 10). One hypothesis was that the formation of 2-oxindole in the monooxygenation reaction catalyzed by MarE might result from the substrate amino group’s distinct orientation, which could prevent it from assisting in the expected epoxide ring opening. However, our structural analysis disproved this hypothesis, leading to the need for further biochemical and spectroscopic studies on the ES complex and reactive intermediates to explore how the structural differences between MarE and TDO affect their reaction outcomes and mechanisms.

### Atypical distal heme ligand geometry

Typically, the heme iron-bound cyanide is positioned perpendicular to the heme plane. However, in our structure, cyanide adopts a bent conformation with an Fe–C–N angle of 131° ± 22° (Fig. S6). The cyanide nitrogen atom interacts with the substrate's amino group and the main chain nitrogen of Gly127 from the DATG loop (Fig. 5). Interestingly, this unusual bent conformation has also been observed in the cyanide-bound hIDO1 structure (25), suggesting that the second coordination sphere influences the distal ligand geometry.

### Conclusive perspectives on MarE oxygenation chemistry

The structural determination of MarE bound to its natural substrate provides a detailed perspective on the histidine-ligated heme prosthetic group, β-Me-l-Trp in the distal heme pocket, and their interactions. The observed enzyme-substrate interaction mode aligns with that of TDO, suggesting the involvement of additional factors in governing reaction specificity and control. Our study identifies key structural distinctions between MarE and TDO that likely underpin their divergent oxygenation chemistries. Despite their evolutionary and structural closeness as members of the HDAO superfamily, MarE and TDO exhibit markedly different oxygenation outcomes on the tryptophan indole ring. The individual and cooperative structural differences highlighted here may contribute to these distinct outcomes, particularly at intermediary stages of the oxygenation process. Further exploration of these factors is crucial to fully elucidate the mechanistic basis of their functional divergence.

## Experimental procedures

### Preparation of **β**-Me-l-Trp

Synthesis of β-Me-l-Trp was performed as previously described (19). The *Pf*TrpB^2B9^ plasmid used for expressing the TrpB protein was generously shared by the Arnold Lab (19). The purified *Pf*TrpB^2B9^ protein was concentrated to 650 μM for storage at −80 °C until use. Briefly, a 20 ml vial was charged with indole (63.2 mg, 0.54 mmol) and l-threonine (595.8 mg, 5 mmol). The solid mixture was suspended with 3.5 ml of a 5% DMSO buffer solution containing potassium phosphate (200 mM, pH 8.0). A 15 mM stock of pyridoxal phosphate was made in water and added to the mixture to a 100 μM final concentration. Purified *Pf*TrpB^2B9^ was added to a final concentration of 160 μM to a final total volume of 5 ml, and the reaction vial was heated to 75°C in an oil bath while stirring. After 18 h, the reaction was removed from heat, cooled to room temperature, and the solids pelleted by centrifugation.

β-Me-l-Trp was purified from the crude supernatant using a Teledyne ISCO Combiflash Rf system equipped with a RediSep Gold C18 column. The column was washed with 2 volumes of H_2_O before an isocratic elution using 50% methanol/H_2_O. The solvent was removed under reduced pressure, and a lightly tan solid was recovered with a 63.4% yield. Please see Figs. S2 and S3 for the high-resolution mass spectrometry and ^1^H-NMR spectra of the purified β-Me-l-Trp. ^1^H-NMR (300 MHz, D_2_O) δ 7.67 (dt, *J* = 8.0, 1.1 Hz, 1H), 7.45 (dt, *J* = 8.2, 1.0 Hz, 1H), 7.27 (s, 1H), 7.19 (ddd, *J* = 8.2, 7.0, 1.3 Hz, 1H), 7.09 (ddd, *J* = 8.1, 7.0, 1.2 Hz, 1H), 3.86 (d, *J* = 6.6 Hz, 1H), 3.63 (p, *J* = 7.1 Hz, 1H), 1.46 (d, *J* = 7.3 Hz, 3H).

### Cloning, expression, and purification of MarE

A codon-optimized full-length (1–284) gene of MarE from *Streptomyces* sp. B9173 (UniProt ID: X2D878) was synthesized and purchased from GenScript. The synthesized MarE gene was introduced into an expression vector pET-28aTEV using NdeI and HindIII sites. The vector pET-28aTEV is a modified version of the original pET-28a (Merck) in which the thrombin cleavage site has been replaced with a Tobacco etch virus (TEV) protease cleavage site. This modification is designed to facilitate the removal of the N-terminal polyhistidine tag.

To generate C280S variant, 5′-GTGGCGGTAACGGTAGCCCGGTTCAGCAC-3′ was designed as the following forward primer. The reverse complement sequence of the forward primer was used as the reverse primer. Δ(270–284) truncated variant was generated using forward primer, 5′-TAAAAGCTTGCGGCCGC-3′ and reverse primer, 5′-GGTGTCATGACGGGTAC-3′. For site-directed mutation, 5′- TTCTTTCAAGTTACCGCCCTGATCACCGAATA-3′ for H55A, 5′- TTCTTTCAAGTTACCTTCCTGATCACCGAATA-3′ for H55F, 5′- TGCTGATGATGGCTAACGCGCTGC-3′ for R118A, 5′- CTGCTGATGATGAAGAACGCGCTGCCG-3′ for R118K were designed as forward primers with its reverse complement pairs. The DNA sequences were verified by DNA sequencing (Eurofins Genomics).

His-tagged MarE proteins were expressed in *E. coli* BL21 (DE3). The bacterial cell culture was carried out using Luria Bertani (LB) medium at 37 °C with shaking at 220 rpm. The gene expression was induced by supplementing 0.5 mM isopropyl-l-thio-β-D-galactopyranoside (IPTG) when the optical density at 600 nm (OD_600_) reached 0.8. Cells were harvested after 16 to 20 h at 20 °C, and suspended with 10 ml of buffer A containing 50 mM Tris-HCl, 200 mM NaCl, 5% glycerol (pH 8.0) per gram of wet biomass. Cell membrane was disrupted by sonicator (Thermo Fisher). A 1-h pulse-on time with 1-s pulse on/1 s pulse off was applied for 300 ml of cell suspension with stirring in wet ice. After removing cell debris by centrifuge at 34,000*g* for 1 h at 4 °C, the supernatant containing His-tagged MarE was purified by immobilized metal affinity chromatography (IMAC) using HisTrap column (Cytiva), which had been equilibrated with buffer A. His-tagged protein was eluted with buffer B (buffer A + 500 mM imidazole). The N-terminal His-tag was cleaved off by treating TEV protease to the IMAC elution fraction during dialysis with buffer C containing 50 mM Tris-HCl, 50 mM NaCl, 10 mM β-mercaptoethanol (pH 7.5) at 4 °C overnight (Fig. S17). His-tag removed MarE protein, which was separated and collected by flowing through the Hestra column using buffer A/B. The flow-through fraction was concentrated and desalted using HiTrap Desalting column (Cytiva) with 50 mM HEPES-NaOH, 50 mM NaCl, 5% glycerol (pH 7.5). The buffer-exchanged untagged MarE protein was used for heme reconstitution. The extinction coefficient at 280 nm (ε_280_) for the untagged full-length MarE protein was calculated as 46,410 M^−1^ cm^−1^ with 31,996.77 Da using Expasy ProtParam tool (https://web.expasy.org/protparam/). The ε_280_ for the untagged MarE Δ(270–284) is identical to the untagged full-length protein but with a reduced molecular weight of 30,551.19 Da.

### Heme reconstitution of MarE

A fresh stock solution of 4 mM hemin chloride was prepared by dissolving it in 50 mM NaOH. This solution was then diluted to a final concentration of 60 μM hemin chloride and mixed with 50 μM of untagged MarE protein in a 1.2:1 hemin to protein ratio, with gentle stirring. The reconstitution process was conducted at room temperature for 3 h, followed by an overnight incubation at 4 °C. The mixture was then centrifuged at 34,000*g* for 10 min at 4 °C to remove any precipitates. The supernatant was concentrated and desalted using a buffer containing 50 mM HEPES, 50 mM NaCl, and 5% glycerol (pH 7.5) to eliminate excess hemin. The heme-reconstituted protein was further concentrated and stored at −80 °C. Through heme reconstitution, MarE reached a heme occupancy of at least 70%, as determined by the heme extinction coefficient at 405 nm (ε_405_ = 169,198 M^−1^ cm^−1^) obtained using the pyridine hemochromagen method (Fig. S1*A*).

The heme concentration was determined by pyridine hemochromagen assay with ε_557_ = 34 mM^−1^ cm^−1^ for the reduced pyridine hemochromagen (18).

### Catalytic competence

Enzyme reactions were prepared using combinations of MarE or its variants, each containing 50 μM heme, 20 mM sodium ascorbate, and 1 mM β-Me-l-Trp in buffer A. These reactions were conducted in a total volume of 250 μl at room temperature for 16 h. The reactions were then halted by filtration through a 10 kDa molecular weight cut-off (MWCO) centrifugal filter (Merck Millipore).

### High-performance liquid chromatography coupled with mass spectrometry (LC-MS)

Filtered reaction mixtures were analyzed using an Ultimate- 3000SD HPLC system coupled with a photodiode array detector and an ISQ EC mass spectrometer (Thermo Scientific). A 20 μl of each sample was injected onto an InertSustain C18 column (4.6 I.D. × 100 mm, 5 μm particle size) from GL Sciences Inc. Isocratic elution was employed, using a mobile phase composed of water, acetonitrile, and 0.1% formic acid. The mobile phase was delivered at a flow rate of 1.0 ml/min. For the analysis of β-Me-l-Trp, a mobile phase containing 6.0% acetonitrile was used. Each chromatographic run lasted 20 min.

### High-resolution mass spectrometry (HRMS)

High-resolution mass spectra were obtained using a maXis plus quadrupole-time of flight mass spectrometer with an electrospray ionization source (Bruker Daltonics), operating in positive ionization mode. LC fraction samples were introduced at a steady flow rate of 3 μl/min *via* a syringe pump. Key source parameters included a capillary voltage of 3500 V, an endplate offset of −500 V, a nebulizer gas pressure of 0.4 bar, a dry gas flow rate of 4.0 L/min, and a source temperature of 200 °C. The mass spectra were averaged over 1 minute of scans, collected at a rate of one scan per second, within the m/z range of 50 to 1500. All mass spectra were processed using Compass Data Analysis software version 4.3 (Bruker Daltonics).

### Crystallization, data collection, and structure determination

For crystallization, untagged full-length MarE C280S variant and untagged MarE Δ(270–284) were utilized. The final polishing step involved gel filtration chromatography using a Superdex 200 column (Cytiva) with isocratic elution in a buffer containing 50 mM HEPES sodium and 200 mM arginine (pH 7.5) for MarE C280S. The untagged and heme-reconstituted MarE C280S was concentrated to 1.99 mM, as determined by absorbance at 280 nm, and with 1.52 mM heme (76.4% heme occupancy), based on absorbance at 405 nm. Sodium cyanide was first added to MarE C280S, followed by β-Me-l-Trp. The final concentrations of MarE C280S heme, sodium cyanide, and β-Me-l-Trp were 250 or 500 μM, 50 mM, and 10 mM, respectively. This mixture was used for crystallization setup. Crystals were obtained using the sitting-drop vapor diffusion method at 16 °C (Fig. S18). The protein drop (1–2 μl) was mixed with an equal volume of crystallization solution containing 18% Tacsimate (pH 6.0) and 20% (w/v) polyethylene glycol 3350.

For MarE Δ(270–284), 50 mM HEPES-NaOH buffer (pH 7.5) containing 50 mM NaCl was employed for Superdex 200 purification. MarE Δ(270–284) at 1.5 to 1.8 mM concentration incubated with 10 mM β-Me-l-Trp was used to set up crystallization. Crystals were grown by the sitting-drop vapor diffusion method at 22 °C. A 1 μl protein solution was mixed with an equal volume of a reservoir solution containing 25% ethylene glycol.

Single wavelength data were collected at beamline BL9-2 at Stanford Synchrotron Radiation Lightsource (SSRL) (Table 1). X-ray diffraction data were collected at 100 K and processed using the program HKL3000 (44). Ethylene glycol (25–30% v/v) was used as a cryoprotectant to prevent diffraction data loss due to ice rings. The initial phases were obtained by conducting molecular replacement with an AlphaFold predicted structure as a search probe. Iterative model building, inspection and refinement were performed using COOT (45) and PHENIX (46). X-ray data collection and structure refinement statistics are summarized in Table 1. Ramachandran plot analysis were performed using MolProbity (47). Structural visualizations were generated using PyMOL (Schrödinger, version 2.3.3). Multiple sequence alignments were conducted with Clustal Omega (48) and visualized using ESPript (49). Structural superpositions of MarE with structurally validated HDAO members were carried out using CCP4MG (50).

### Isothermal titration calorimetry (ITC)

A Microcal VP-ITC system (Malvern Instruments) was employed to conduct isothermal titration calorimetry (ITC) experiments. Protein samples were buffer-exchanged using HiTrap Desalting column (Cytiva) equilibrated with a buffer containing 50 mM HEPES sodium, 50 mM NaCl, and 5% glycerol (pH 7.5). Protein samples with a heme concentration of 0.1 mM were titrated with either 1.25 mM or 2.5 mM β-Me-l-Trp in the presence of 0.1 mM sodium cyanide. A total of 29 injections were performed at 22 °C, with a reference power of 30 μcal/s and a stirring speed of 155 rpm. Data processing and analysis were carried out using Origin version 7.0 (OriginLab Corp). Dissociation constants (*K*_D_) were determined by a non-linear curve fitting to a one-site binding model. ITC measurements were performed in triplicate following our previously published protocol (51) and the ITC parameters (N, *K*_D_, ΔH, and ΔS) were reported as averaged values with errors in standard deviation.

## Data availability

The crystal structure has been deposited in the RCSB Protein Data Bank with the PDB entries 8VYY and 9CA3. All other data are contained in the article and Supporting Information.

## Supporting information

This article contains supporting information (Figs. S1–S18).

## Conflict of interest

The authors declare that they have no conflicts of interest with the contents of this article.
