# Supplementary material for: Structural insights into 2-oxindole-forming monooxygenase MarE: Divergent architecture and substrate positioning versus tryptophan dioxygenases
Source: J Biol Chem. 2025 Jan 27;301(3):108241. doi: 10.1016/j.jbc.2025.108241 (PMC11904535; doi:10.1016/j.jbc.2025.108241)
Supplement: Supporting information [file mmc1.pdf]

Supporting Information

Structural insights into 2-oxindole-forming monooxygenase MarE: Divergent architecture and substrate positioning versus tryptophan dioxygenases

Inchul Shin, Romie C. Nguyen, Samuel R. Montoya, and Aimin Liu*

From the Department of Chemistry, The University of Texas at San Antonio, Texas 78249, United States

Running title: *Crystal structure of oxindole-forming enzyme*

*To whom correspondence may be addressed: Aimin Liu (Feradical@utsa.edu)

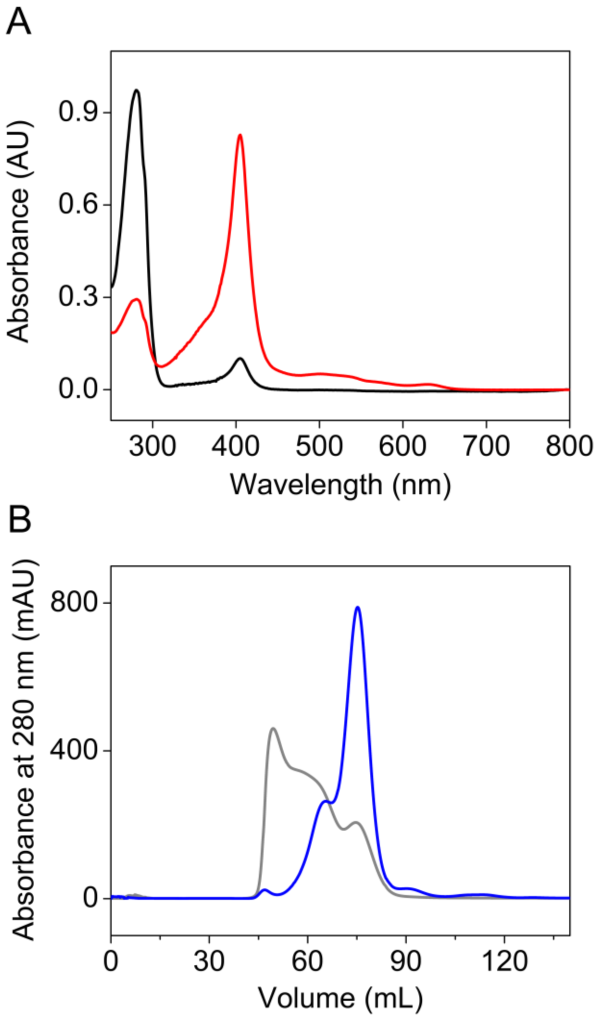

Figure S1. Improvement of heme occupancy and protein homogeneity for MarE crystallization. *A*, The heme-reconstitution reaction substantially improved the heme occupancy of MarE. UV-visible spectra before and after the heme-reconstitution reaction are shown in the black and red traces, respectively. Heme occupancy was increased from 3% (black) to 80% (red). *B*, Improvement in gel-filtration chromatography pattern from a Superdex-200 column after introducing C280S mutation in MarE. Gray and blue traces represent the tag-free and heme-reconstituted wildtype and C280S variant of MarE, respectively.

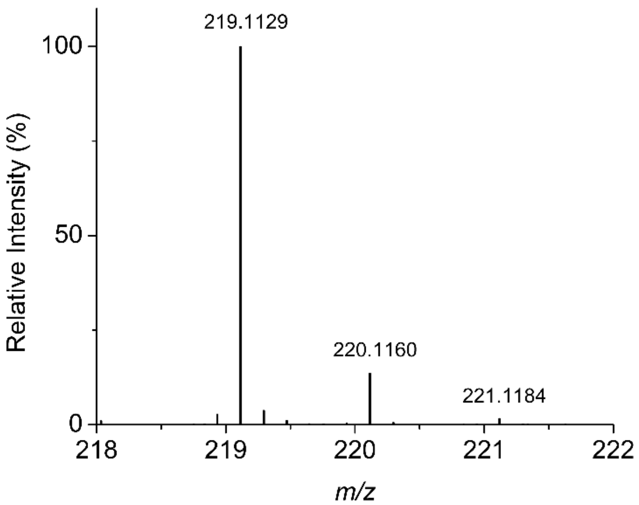

Figure S2. HRMS spectra of β-Me-L-Trp. $[M + H]^+$ calculated for $C_{12}H_{14}N_2O_2$ = 219.1128, $[M + H]^+$ observed = 219.1129, Error = 0.46 ppm.

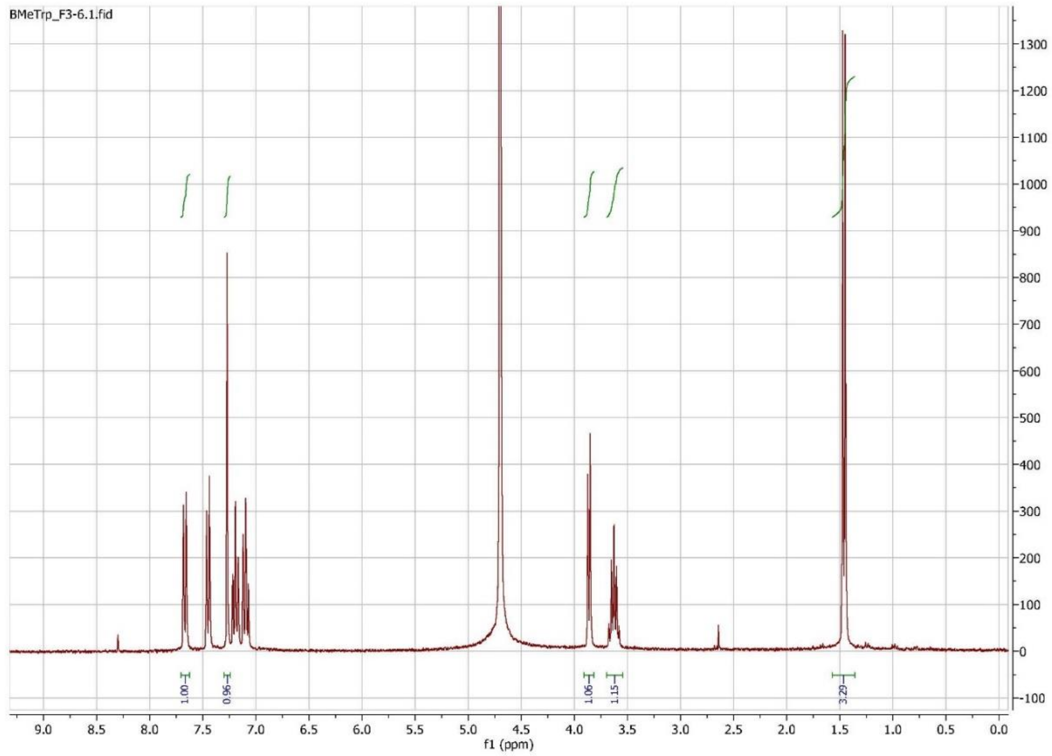

Figure S3. ^{1}H-NMR spectra of β-Me-L-Trp.

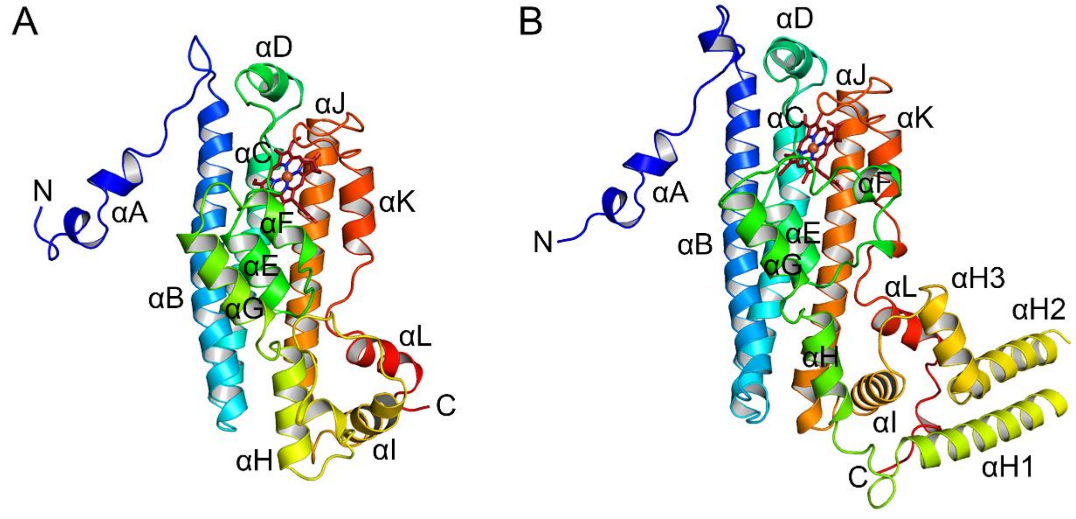

Figure S4. Monomeric structures of XcTDO and hTDO. Monomeric subunits of (A) XcTDO (PDB entry: 2NW8) and (B) hTDO (PDB entry: 5TIA) are presented in rainbow color scale from blue to red color denoting N- to C- terminus. Heme is presented in a red stick model.

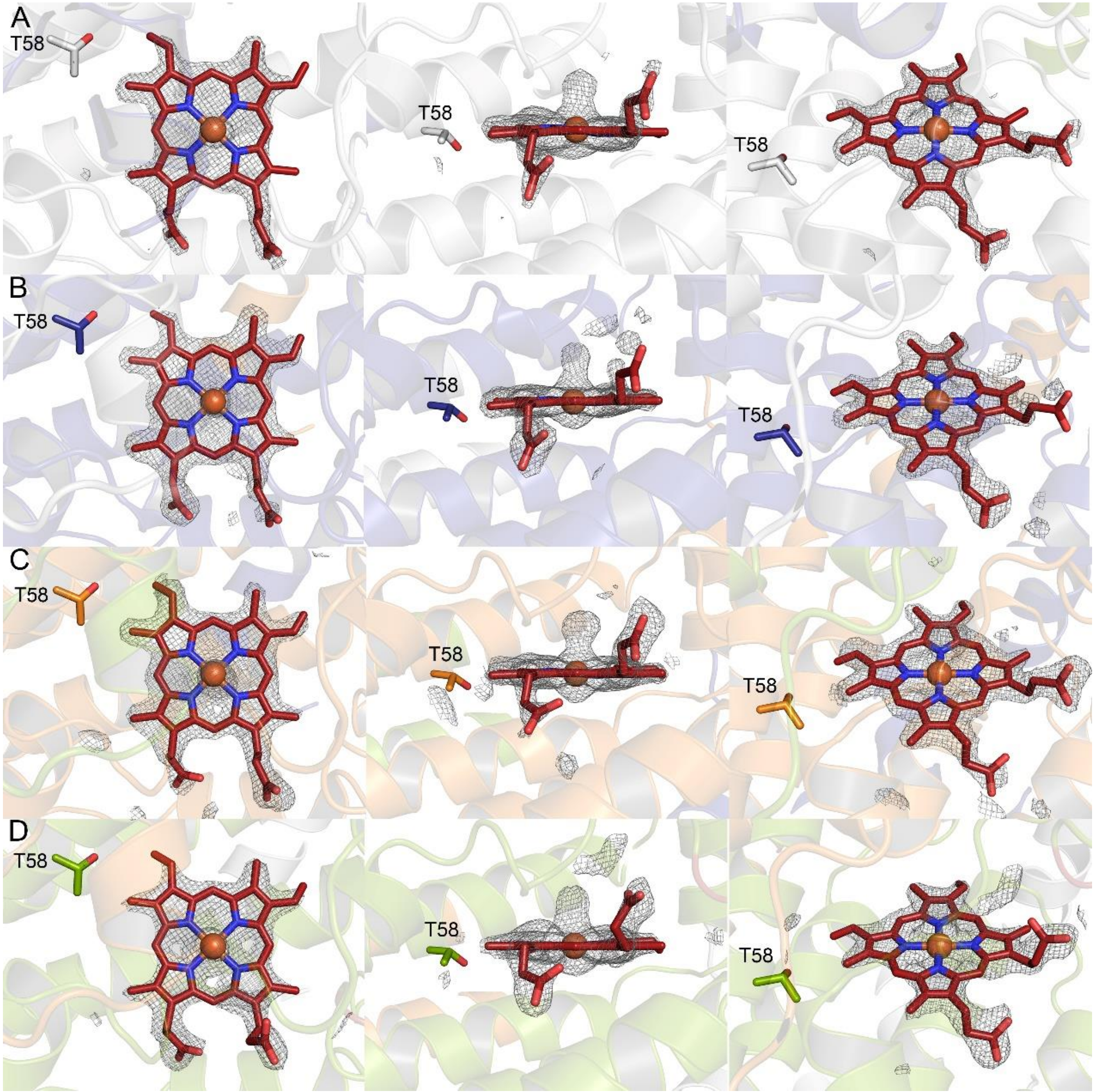

Figure S5. Electron density for heme. From left, top, front, and side view of heme with omit F_o–F_c map contoured at 3 σ in subunit A, B, C, and D.

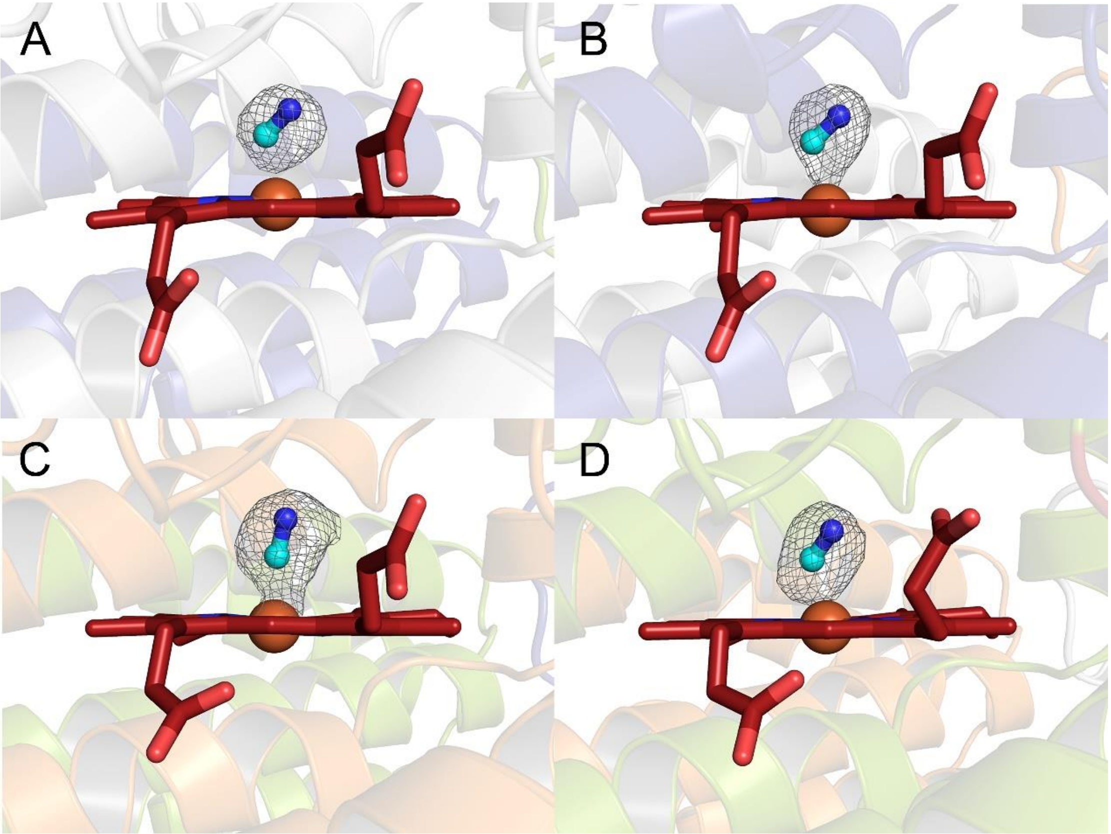

Figure S6. Electron density for cyanide. Omit F_o-F_c maps contoured at 3 σ in subunits A, B, C, and D. Bond between the carbon atom of cyanide (cyan color) and the iron ion is omitted for clarity. The lengths of Fe–C bond and the angles of Fe–C–N in each subunit are: 2.12 Å, 120.83°; 2.12 Å, 119.10°; 2.11 Å, 163.52°; 2.12 Å, 120.44°.

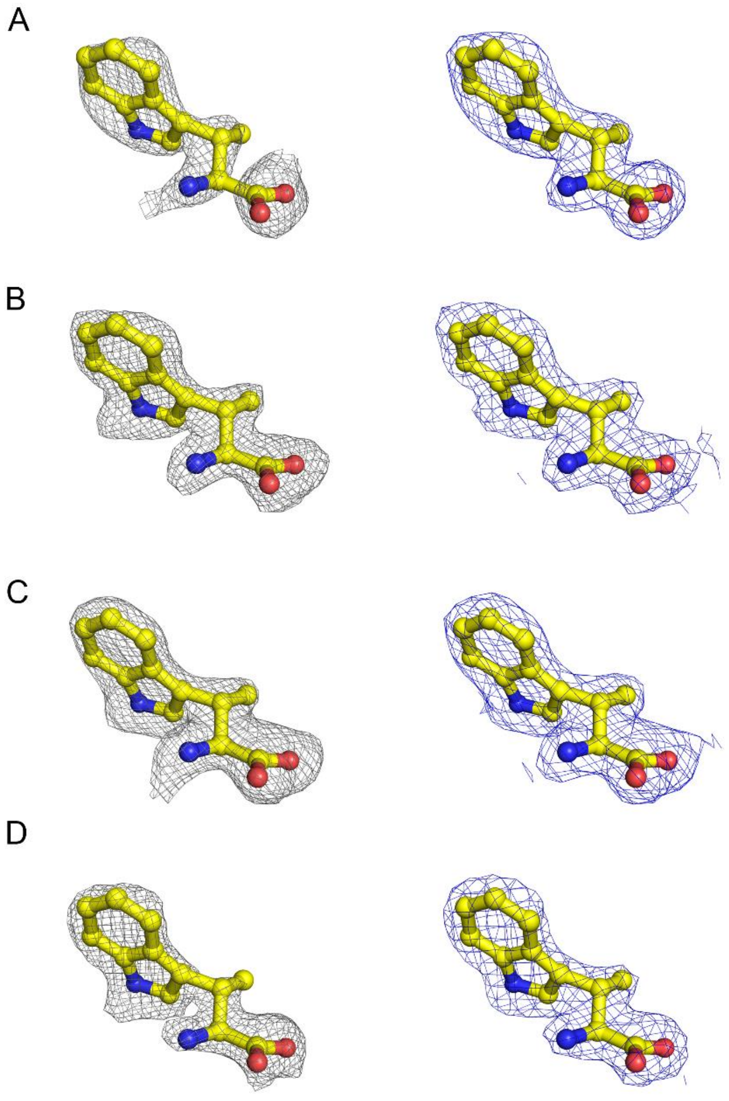

Figure S7. Electron density maps for β-Me-L-Trp. The omit F_o–F_c maps (gray) contoured at 3 σ and $2F_o$–F_c maps (blue) contoured at 1 σ for β-Me-L-Trp in each subunit are shown.

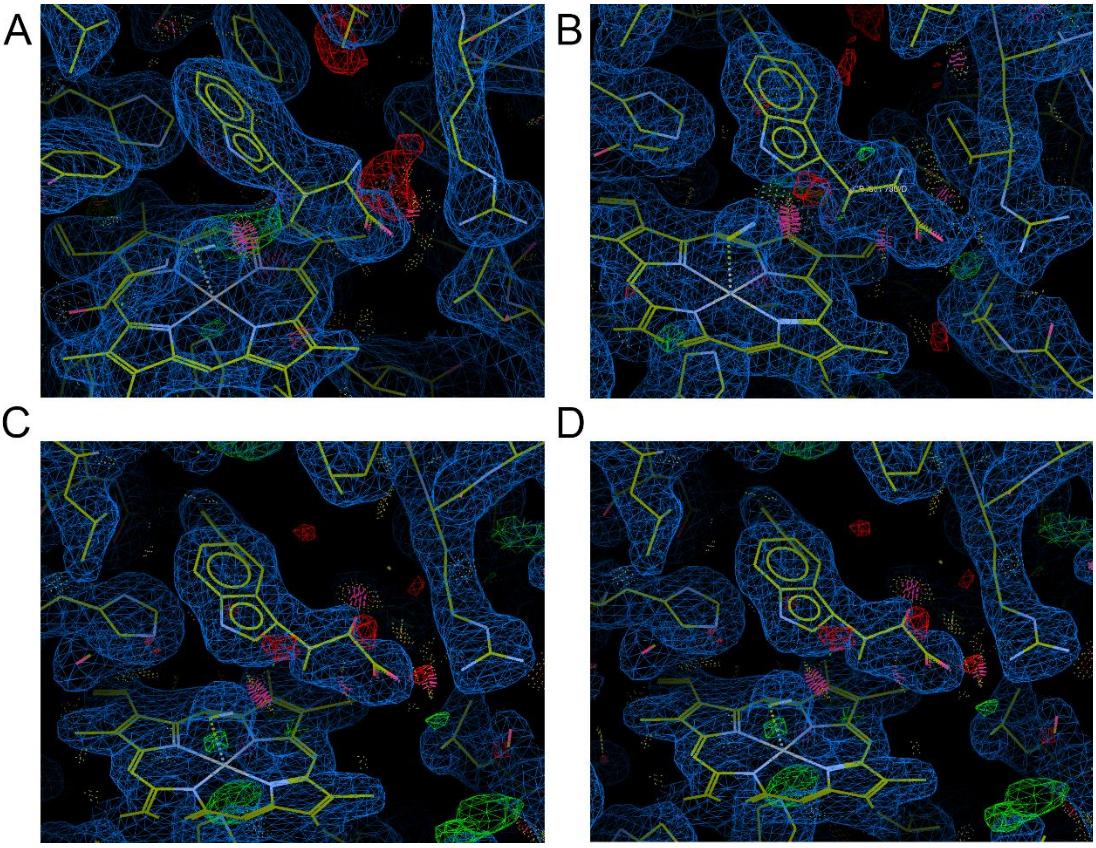

Figure S8. Attempt to fit β-Me-L-Trp into the electron density in a different orientation. Structural refinement was performed after fitting β-Me-L-Trp as the β-methyl group facing toward the heme center and the amino group pointing away from the heme center. Refinement results are shown for each subunit.

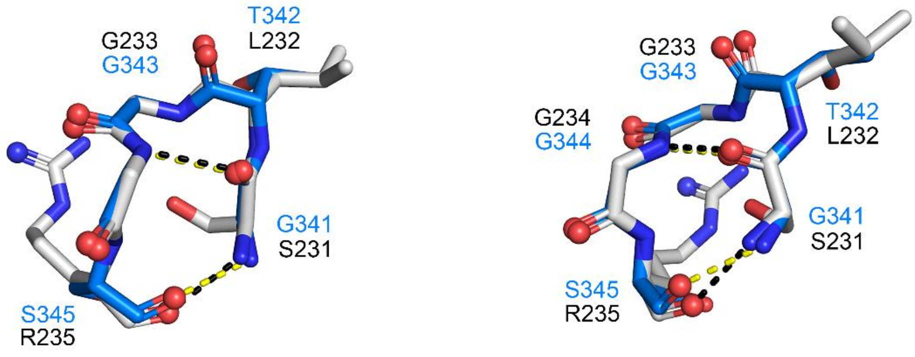

Figure S9. Superposition of SLGGR of MarE on GTGGS of hTDO. SLGGR of MarE and GTGGS of hTDO are presented in gray and blue carbon color with two different orientations. Black and yellow dotted lines are for SLGGR of MarE and GTGGS of hTDO.

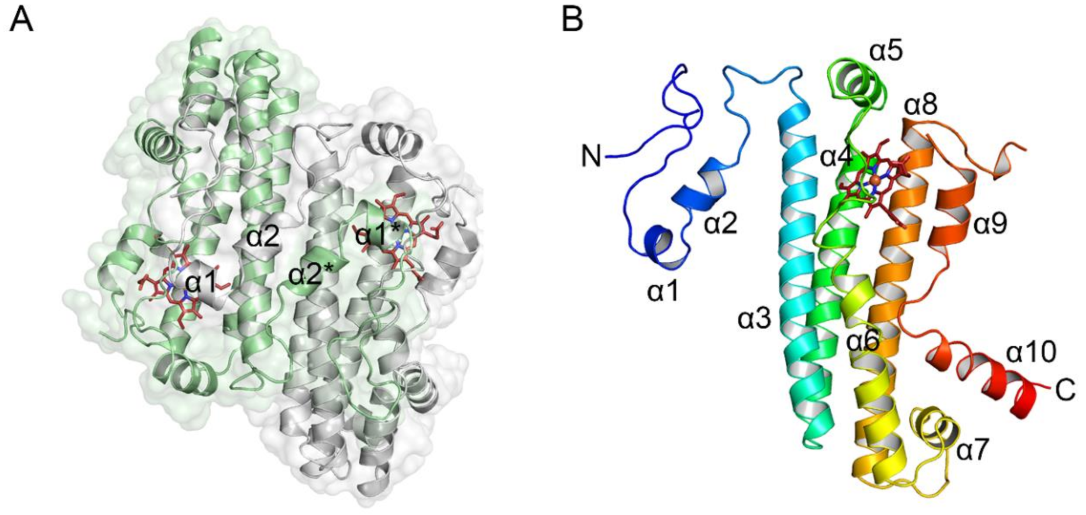

Figure S10. Crystal structure of MarE Δ(270-284). *A*, Overall crystal structure of MarE Δ(270-284) is shown. The asymmetric unit contains a pair of MarE monomers colored in gray and light green. Heme is presented in a red stick model. *B*, Monomeric structure of MarE Δ(270-284). MarE Δ(270-284) monomer structure is shown in rainbow color scale from blue to red, denoting N- to C-terminus. Heme is presented in a red stick model. Residues ranging from 217 – 234 are disordered between helices α8 and α9.

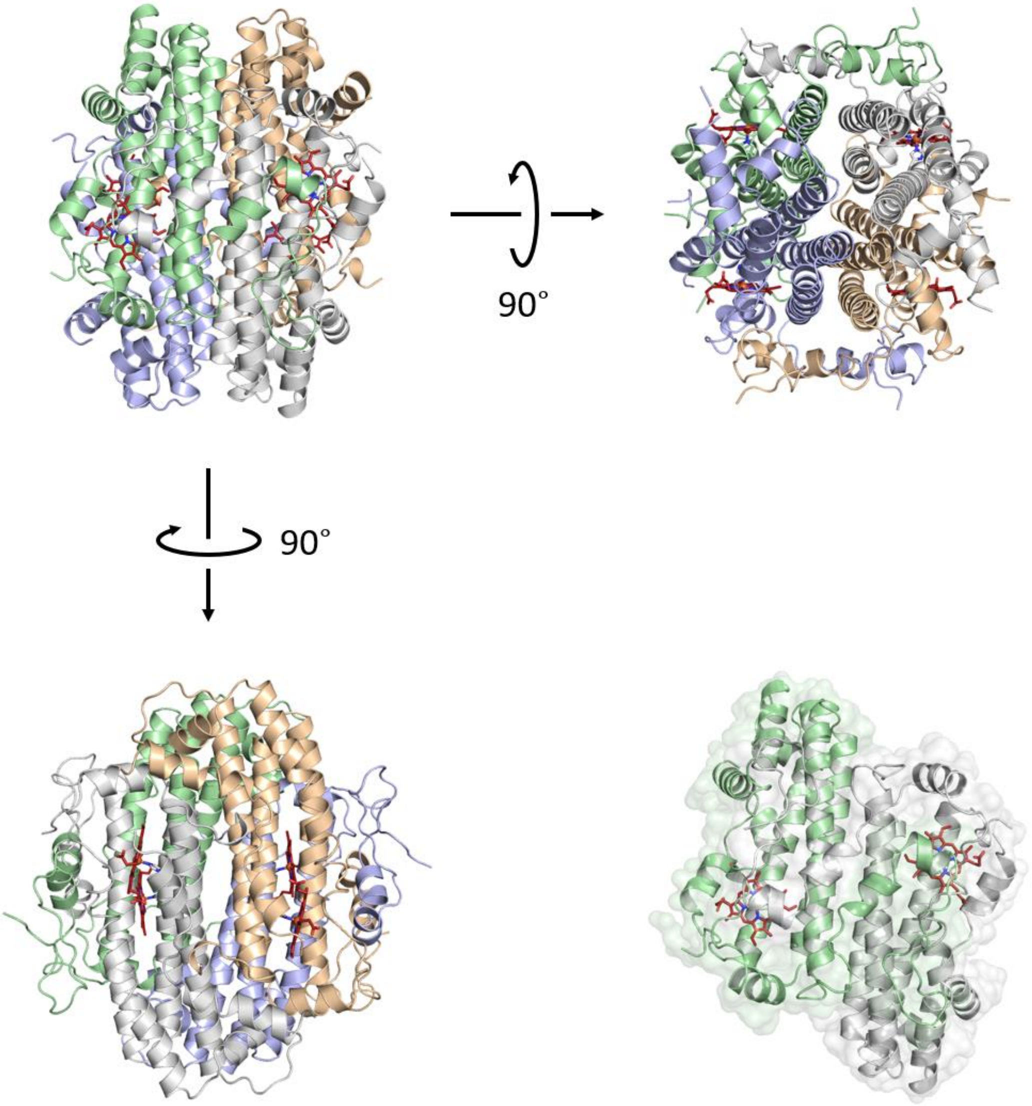

Figure S11. Homotetrameric structure of MarE Δ(270-284). The asymmetric unit contains two protomers, gray and light green (bottom right). The tetrameric structure was constructed with crystallographic symmetry-generated pairs, as in wheat and light blue. Heme is presented in a red stick model.

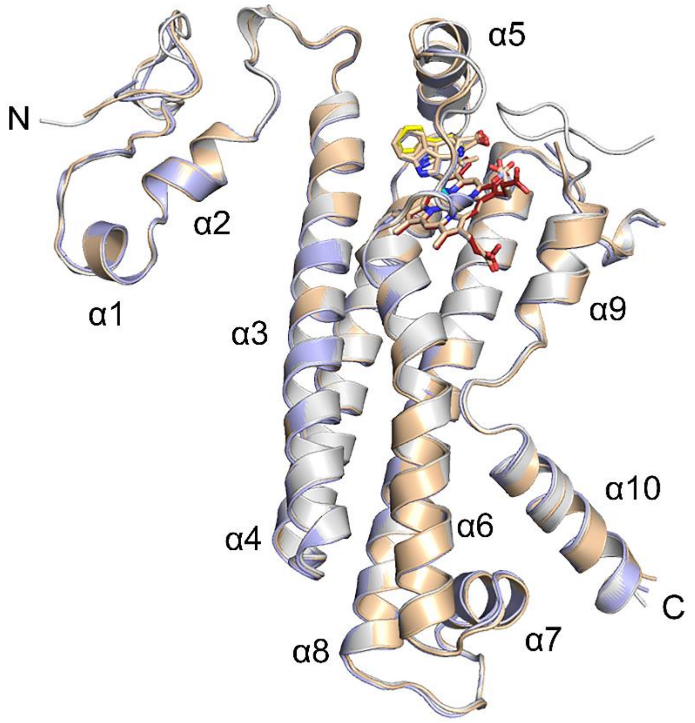

Figure S12. Superposition of MarE Δ(270-284) on the ternary complex structure of MarE C280S. Chain A of the ternary complex of MarE C280S is shown in gray with a red heme stick model. Chain A and chain B of MarE Δ(270-284) are shown in wheat and light blue colors, respectively. Both MarE Δ(270-284) chains are nearly identical to chain A of the ternary complex of MarE C280S with an RMSD value of 0.54 Å for 243 Cα atoms.

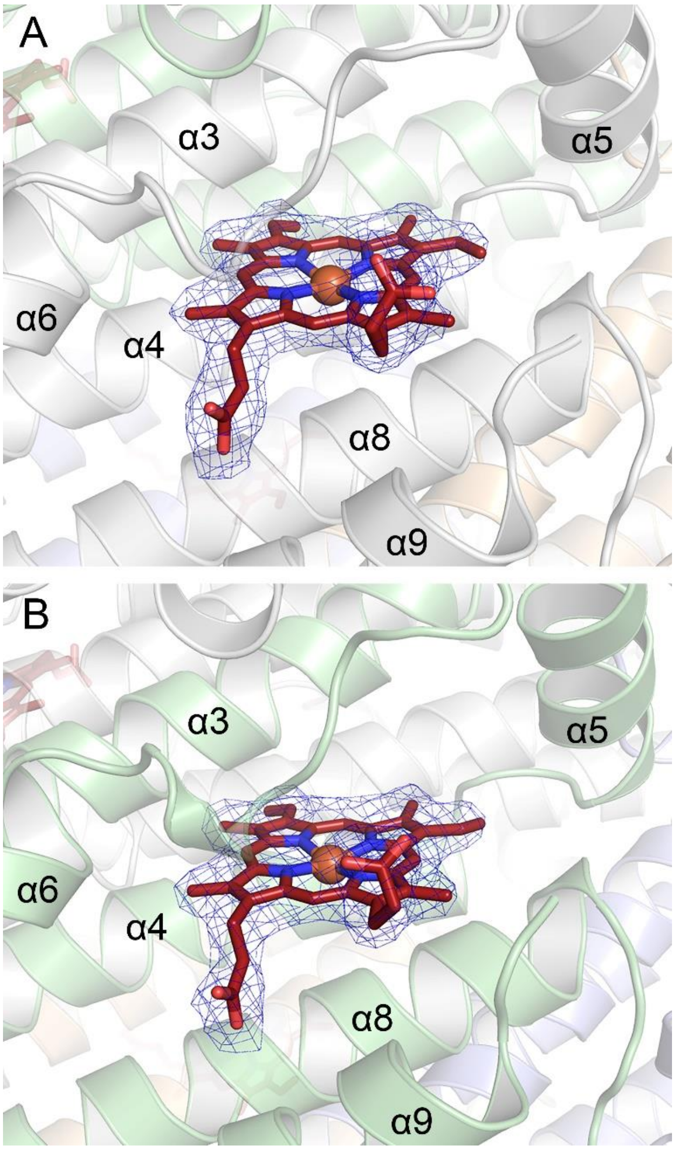

Figure S13. Heme electron density of MarE Δ(270-284). *A*, Heme electron density in chain A (gray). *B*, Heme electron density in chain B (light green). Heme electron densities are shown in a blue $2F_o–F_c$ omit map contoured at 0.8 σ.

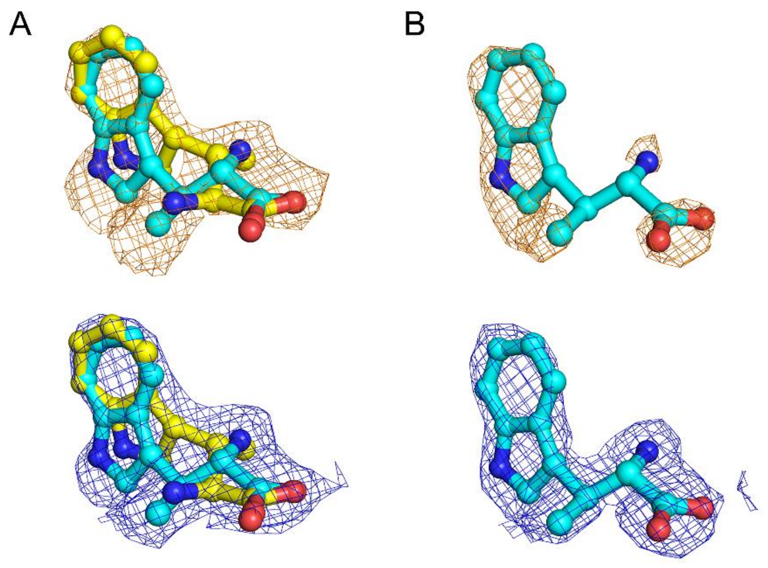

Figure S14. Electron density for β-Me-L-Trp bound in the binary complex structure of MarE Δ(270-284). *A*, Omit F_o–F_c map (orange cage), and $2F_o$-F_c map (blue cage) in chain A are shown. *B*, Omit F_o-F_c map (orange cage) and $2F_o$-F_c map (blue cage) in chain B are shown. Conformation A and conformation B of β-Me-L-Trp are shown in yellow and cyan carbon colors, respectively. Omit F_o–F_c and $2F_o$–F_c maps are contoured at 3.0 σ and 0.8 σ, respectively.

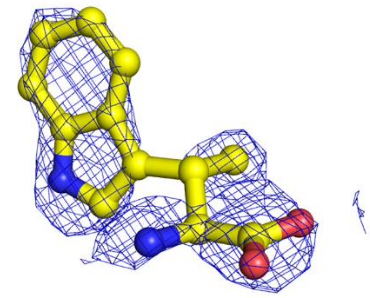

Figure S15. Alternative fitting attempts of β-Me-L-Trp in chain B of the binary complex. IDO/TDO-like conformation A of β-Me-L-Trp in chain B of the binary complex structure of MarE Δ(270-284) shows poor fitting in the electron density map after refinement. Occupancy was set to 0.75 as conformation B in chain B. $2F_o–F_c$ map is shown at 0.8 σ.

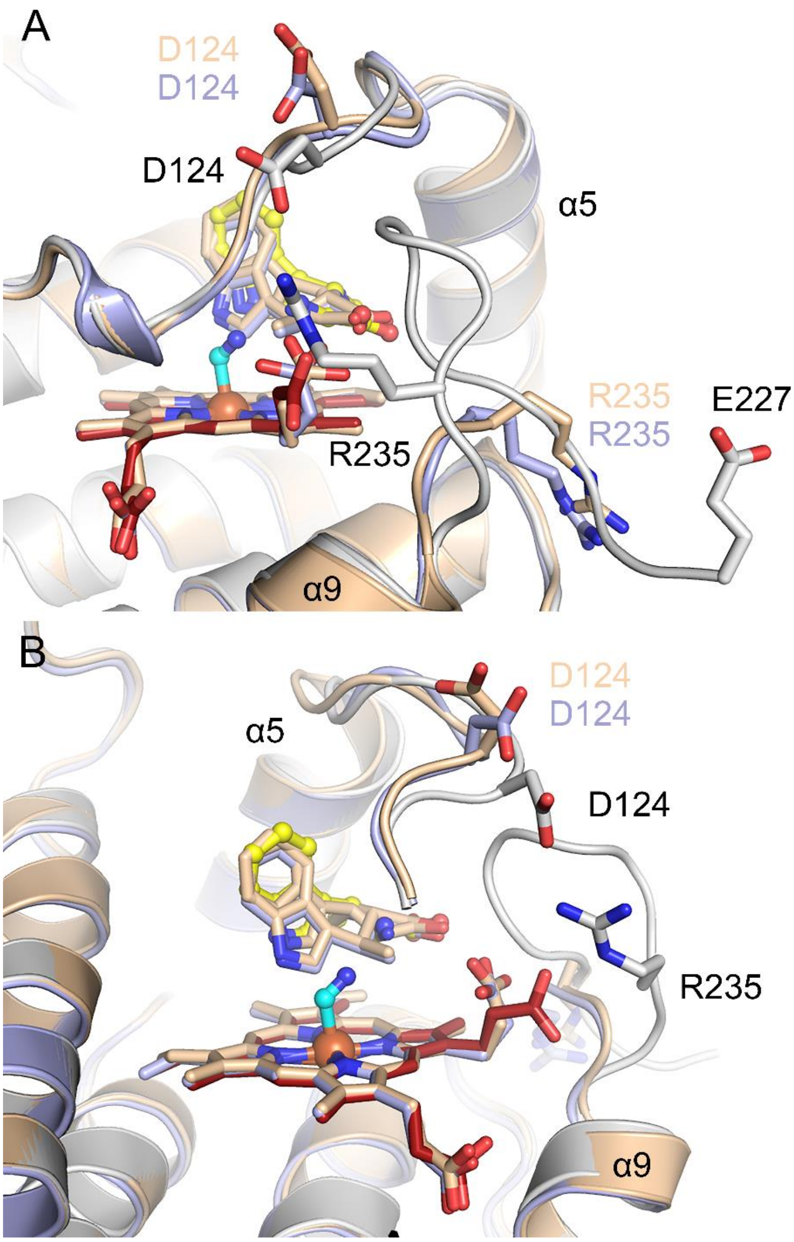

Figure S16. Ternary and binary complex structures show differences in two active site loops. Superposed structures are shown in two orientations, A and B, with wheat, light blue, and gray colors for chains A and B of the binary complex and chain A of the ternary complex, respectively. β-Me-L-Trp, cyanide, and heme of the ternary complex are shown in yellow, cyan, and red stick models. Gly127 to Leu136 were omitted for clarity in panel *B*.

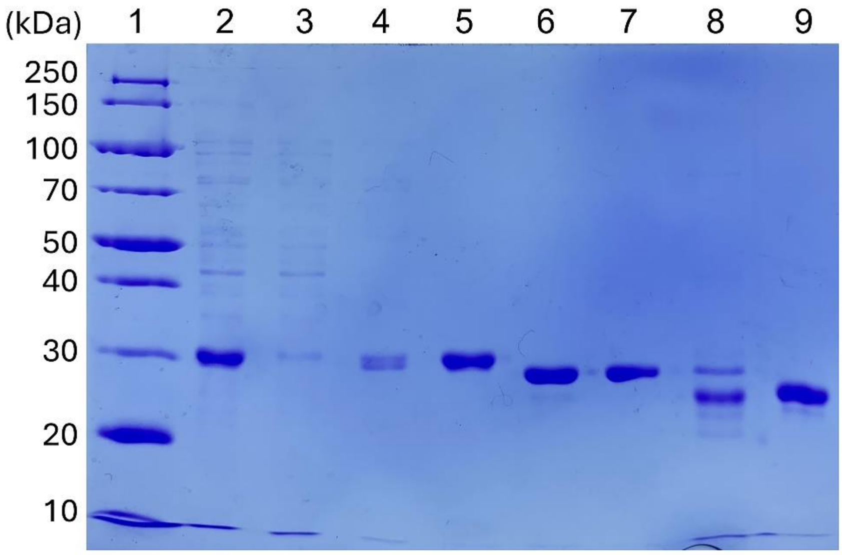

Figure S17. SDS-PAGE confirms N-terminal His-tag removal and its purity. Lane 1, protein size marker (Thermo Fisher #26630); Lane 2, IMAC loading sample of MarE C280S; Lane 3, IMAC flow-through fraction; Lane 4, IMAC wash fraction; Lane 5, IMAC elution fraction; Lane 6, second IMAC loading sample after treating TEV protease and incubating overnight at 4°C during dialysis against 50 mM Tris-HCl, 50 mM NaCl, 10 mM β-mercaptoethanol pH 7.5; Lane 7, second IMAC flow-through fraction; Lane 8, second IMAC elution fraction with 500 mM imidazole; Lane 9, TEV protease. Note that the bands in lanes 6 and 7 migrated further than those in lane 5, indicating that the N-terminal His-tag cleaved off.

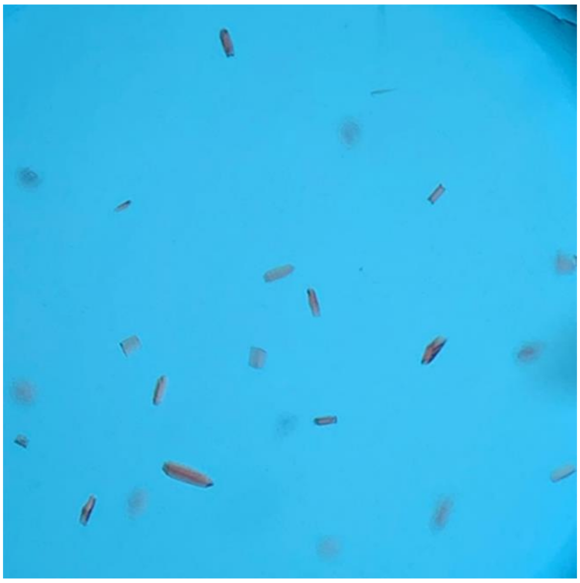

Figure S18. Image of MarE C280S ternary complex crystals.
